# Comprehensive analysis of candidate signatures of long non-coding RNA LINC01116 and related protein-coding genes in patients with hepatocellular carcinoma

**DOI:** 10.1186/s12876-023-02827-y

**Published:** 2023-06-20

**Authors:** Xiang-Kun Wang, Xu-Dong Zhang, Kai Luo, Long Yu, Shuai Huang, Zhong-Yuan Liu, Ren-Feng Li

**Affiliations:** Departments of Hepatobiliary and Pancreatic Surgery, The First Affiliated Hospital of Zhengzhou University, Henan Province, Zhengzhou, 450052 P. R. China

**Keywords:** Long non-coding RNA, LINC01116, Protein-coding gene, Signature, Hepatocellular carcinoma

## Abstract

**Background:**

Hepatocellular carcinoma (HCC) is a long-term malignancy that causes high morbidities and mortalities worldwide. Notably, long non-coding RNAs (LncRNAs) have been identified as candidate targets for malignancy treatments.

**Methods:**

LncRNA LINC01116 and its Pearson-correlated genes (PCGs) were identified and analyzed in HCC patients. The diagnostic and prognostic value of the lncRNA was evaluated using data from The Cancer Genome Atlas (TCGA). Further, we explored the target drugs of LINC01116 for clinical application. Relationships between immune infiltration and PCGs, methylation and PCGs were explored. The diagnostic potentials were then validated by Oncomine cohorts.

**Results:**

LINC01116 and the PCG *OLFML2B* are differentially and highly expressed in tumor tissues (both *P* ≤ 0.050). We found that LINC01116, *TMSB15A*, *PLAU*, *OLFML2B,* and *MRC2* have diagnostic potentials (all AUC ≥ 0.700, all *P* ≤ 0.050) while LINC01116 and *TMSB15A* have prognostic significance (both adjusted *P* ≤ 0.050). LINC01116 was enriched in the vascular endothelial growth factor (VEGF) receptor signaling pathway, mesenchyme morphogenesis, etc. After that, candidate target drugs with potential clinical significance were identified: Thiamine, Cromolyn, Rilmenidine, Chlorhexidine, Sulindac_sulfone, Chloropyrazine, and Meprylcaine. Analysis of immune infiltration revealed that *MRC2*, *OLFML2B*, *PLAU*, and *TMSB15A* are negatively associated with the purity but positively associated with the specific cell types (all *P* < 0.050). Analysis of promoter methylation demonstrated that *MRC2*, *OLFML2B*, and *PLAU* have differential and high methylation levels in primary tumors (all *P* < 0.050). Validation results of the differential expressions and diagnostic potential of *OLFML2B* (Oncomine) were consistent with those obtained in the TCGA cohort (*P* < 0.050, AUC > 0.700).

**Conclusions:**

Differentially expressed LINC01116 could be a candidate diagnostic and an independent prognostic signature in HCC. Besides, its target drugs may work for HCC therapy via the VEGF receptor signaling pathway. Differentially expressed *OLFML2B* could be a diagnostic signature involved in HCC via immune infiltrates.

**Supplementary Information:**

The online version contains supplementary material available at 10.1186/s12876-023-02827-y.

## Introduction

Hepatocellular carcinoma (HCC) is the sixth most prevalent form of malignancy worldwide and the second leading cause of tumor-related deaths [1]. China alone accounts for approximately 55% of the global HCC cases annually due to the chronic hepatitis B virus and liver cirrhosis [2]. The morbidity and mortality of HCC are particularly high in China [3]. Although many advanced treatments, including surgical resection, liver transplant, and comprehensive therapies, have been in clinical application, the 5-year overall survival (OS) rate of HCC patients is still unsatisfactory [4]. On the other hand, many researches have used big data genomics and molecular biology to identify various carcinogenic factors and molecular modulatory mechanisms of HCC. However, many patients are diagnosed at an advanced stage, and the tumors are prone to recurrence, even after surgery [5, 6]. The identification of novel candidate biomarkers for early diagnosis, prognostic surveillance, and studies on the molecular mechanisms of HCC is, therefore, of significance.

Non-coding RNAs (ncRNAs), including microRNAs and long non-coding RNAs (LncRNA), have been identified as oncogenes and tumor suppressors in various cancer types. Besides, ncRNAs have emerging roles as novel therapeutic targets [7, 8]. LncRNAs are RNA molecules with a length of more than 200 nucleotides and do not code for proteins [9]. They are often aberrantly expressed in various cancers, such as esophageal [10], bladder [11], and prostate [12], where they function as oncogenes or tumor suppressors. For instance, the LncRNA HOTAIR promotes cell migration and invasion by down-regulating the RNA binding motif protein 38 in HCC [13].

The lncRNA LINC01116, also known as TALNEC2, is located in the 2q31.1 region [14]. Previously, we identified LINC01116 via bioinformatic analysis method as a potentially prognostic biomarker in HCC. Haibei Hu et al. demonstrated that LINC01116 is overexpressed in breast cancer, where it is associated with metastasis and is indicative of a poor prognosis [14]. Jingliang Ye et al. found that the expression of LINC01116 is significantly up-regulated in glioma tissues and could serve as both a diagnostic biomarker and a therapeutic target for glioma [15]. They further suggested that LINC01116 modulates tumorigenesis in glioma by targeting the vascular endothelial growth factor (VEGF) through microRNA-31-5p [15]. The study by Jing Wu showed that LINC01116 is overexpressed in oral squamous cell carcinoma and nasopharyngeal carcinoma tissues, and is associated with OS and relapse-free survival rate of head and neck squamous cell carcinoma (HNSCC) [16]. They showed that LINC01116 acts as a cancer-promoting oncogene via epithelial-mesenchymal transition [16]. Nonetheless, the expression of LINC01116 in HCC, as well as its role in the diagnosis, prognosis, and as a potential molecular target for HCC therapy is obscure. Therefore, the present study explored the potential roles of LINC01116 in HCC to provide new insights into its application in HCC.

## Materials and methods

### Data collection and genome-wide analysis to identify LINC01116-correlated mRNAs

The mRNA expression levels and clinical data of patients pathologically diagnosed with HCC were downloaded from The Cancer Genome Atlas (TCGA, https://www.cancer.gov/). We then performed a genome-wide analysis by Pearson correlation analysis to determine LINC01116-related protein-coding genes (PCGs) using the R 3.6.0 platform (https://www.r-project.org/).

### Analysis of expression levels and diagnostic potential

We explored the differential expressions and diagnostic potentials of the LncRNA LINC01116 and its top ten PCGs, as determined by correlation coefficient analysis. We first used the MERAV website (http://merav.wi.mit.edu/) to obtain the differential expression between tumorigenic and normal liver tissues for LINC01116 and 10 PCGs [17]. The differential expressions were then analyzed using tumor and non-tumor data from the TCGA database. After that, we further explored the diagnostic potential of LINC01116 and the ten PCGs using receiver operative characteristic (ROC) curves in the TCGA database.

### Prognostic analysis and conjoint analysis

The mRNA expressions of LINC01116 and the ten PCGs were divided into low and high expression groups by the median cutoff. Clinical data were then analyzed along these associations to assess the OS status. The Kaplan–Meier plot method and Cox hazard regression model were applied for univariate and multivariate analysis, respectively. Clinical factors related to OS were enrolled in the multivariate Cox regression model. The PCGs identified in the multivariate Cox regression model were determined as OS-related genes. Then, LINC01116 and these genes were used for conjoint analysis within their low or high expression groups.

### Construction of predictive model using risk scores and nomogram

A risk score prediction model was constructed to predict patient survival based on its scores. A risk score prediction model was constructed using LINC01116 and OS-related PCGs as follows: risk scores = gene_1_*β_1_ + gene_2_*β_2_ + gene_3_*β_3_ + … + gene_n_*β_n_ [18, 19]. Where β was the coefficient from the multivariate cox regression model, including LINC01116, PCGs, and clinical data. Then, low and high-risk groups were generated from the respective risk scores at the median cutoff. In addition, a nomogram was constructed using LINC01116, PCGs, and clinical data to predict survival probability at 1–5 years. Internal validation using c-index was further performed at 1–5 years.

### Exploration of molecular mechanisms by gene set enrichment analysis (GSEA)

GSEA was performed to explore the potential molecular mechanisms, including gene ontology terms and metabolic pathways of OS-related PCGs and LINC01116. Analysis was conducted using a GSEA software (gsea2-2.2.4, https://www.gsea-msigdb.org/gsea/index.jsp), c2 curated gene sets (c2.cp.kegg.v7.0.symbols.gmt), and c5 gene ontology sets (c5.all.v7.0.symbols.gmt) [20, 21]. Also, a false discovery rate (FDR) ≤ 0.25 was considered a significant enrichment.

### Identification of potential drug targets of LINC01116

Analysis of the potential drug targets was further conducted to explore the clinical applications of LINC01116 for HCC. Then, a differential analysis was undertaken to obtain differentially expressed genes (DEGs) using the *edgeR* package in the R platform [22]. DEGs were further used to obtain target drugs using the connectivity map website (cMAP, https://portals.broadinstitute.org/cmap/#). Negatively related drugs were considered drug targets.

### Analysis of immune infiltration and methylation of PCGs

Analysis of immune infiltration was conducted via the Tumor Immune Estimation Resource database (TIMER, https://cistrome.shinyapps.io/timer/) [23, 24]. Firstly, we analyzed the correlation between gene expression levels of PCGs and the extend of immune infiltration, including B cells, CD4^+^ T cells, CD8^+^ T cells, neutrophils, macrophages, and dendritic cells (This database did not recognize LncRNA). Then, analysis of somatic copy number alterations (SCNA) was performed to determine the correlations between the SNCA of PCGs and immune infiltration. Analysis of promoter methylation was applied to explore the relationships between gene expressions of PCGs and subgroups of clinical data via the UALCAN database (http://ualcan.path.uab.edu/) (This database did not recognize LncRNAs) [25]. Clinical data, such as tumor types, gender, race, and tumor grade, were used for analysis.

### Construction of competing endogenous RNA (ceRNA) network and validation of clinical significance

A ceRNA network was constructed based on negative regulation relationships between mRNA, miRNA, and lncRNA via LnCeVar database (http://ww.bio-bigdata.net/LnCeVar/index.jsp) [26]. We further validated differential expressions and the diagnostic potentials of LINC01116 and PCGs by the oncomine database. The oncomine database was utilized to validate the differential expressions and diagnostic potentials of PCGs using scatter plots and ROC curves (this database did not recognize LncRNA).

### Statistical analysis

Survival analysis, including the Kaplan–Meier, univariate, and multivariate Cox hazard regression model, was conducted using the SPSS statistical software package version 24.0. ROC curves, scatter plots, and survival plots were plotted using the GraphPad software version 8.0. A *P* value of ≤ 0.05 was considered statistically significant.

## Results

### Clinicopathological characteristics of HCC patients and top ten PCGs

**Fig. 1:**
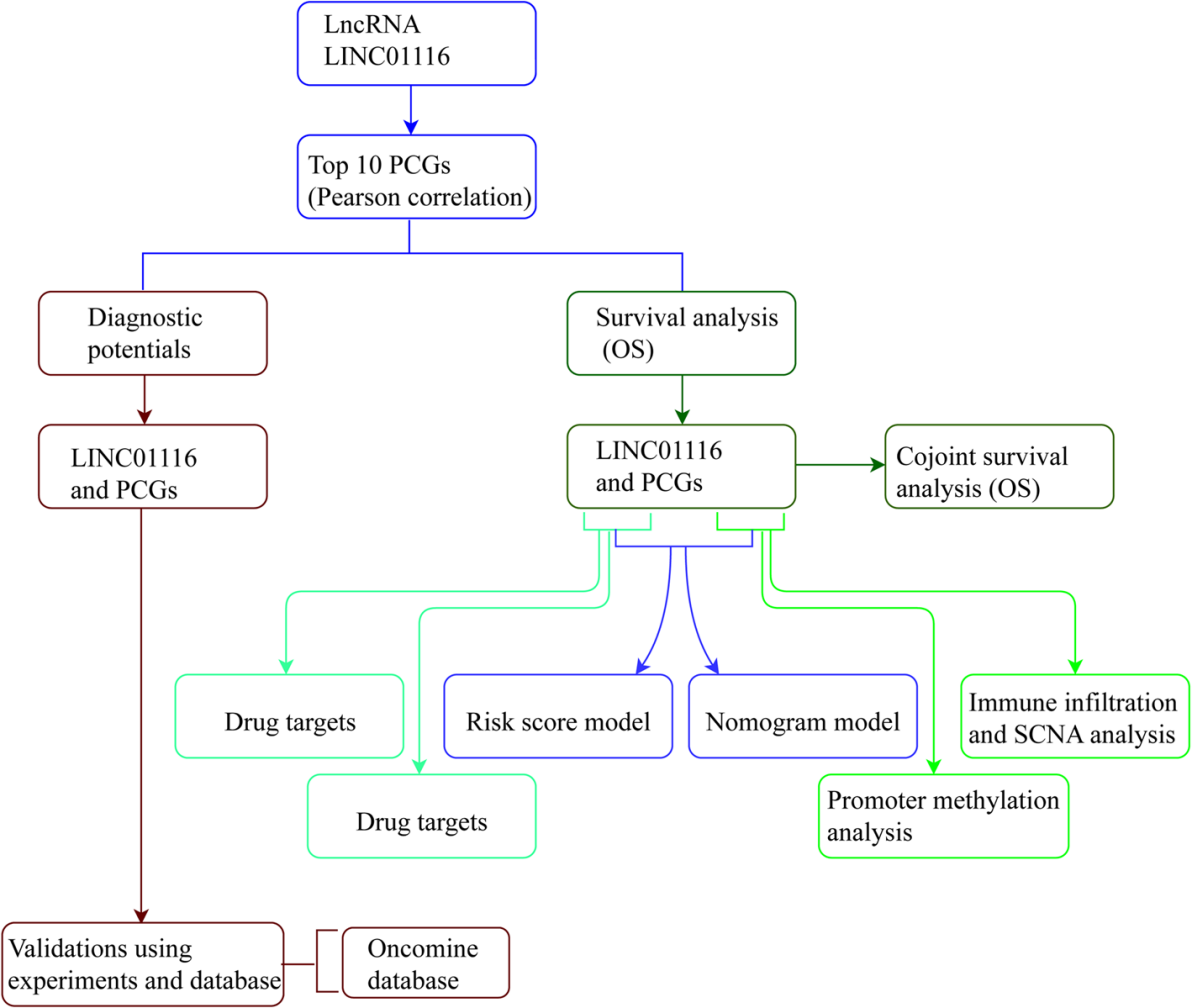
Flow diagram of analysis of LINC01116 and protein-coding genes in HCC

A flow diagram illustrating the process of the present study is shown in Fig. 1. The study included a total of 370 HCC patients, and their clinicopathological characteristics, as obtained in the TCGA dataset, were previously reported [27]. Several factors, including clinical factors, hepatitis B virus (HBV) status, tumor stage, and radical resection, were correlated to the OS (all *P* ≤ 0.05). The top ten PCGs of LINC01116-related by Pearson correlation are as follows: *SOX2*, *BEND6*, *TMSB15A*, *PLAU*, *OLFML2B*, *NTNG1*, *SLC17A7*, *NTRK1*, *MRC2* and *SLC7A3* (all correlation ≥ 0.800, all *P* < 1E-80, Table 1). Following Pearson correlation analysis, the genes associated with LINC01116 are shown in Table S1.

**Table 1:** Top 10 protein-coding genes of Pearson correlation-related with *LINC01116*

LncRNA	PCG	Correlation	95% CI	*P* value
*LINC01116*	*SOX2*	0.820	0.780–0.850	1.87E-89
*LINC01116*	*BEND6*	0.810	0.780–0.850	4.16E-89
*LINC01116*	*TMSB15A*	0.810	0.770–0.840	4.18E-88
*LINC01116*	*PLAU*	0.810	0.770–0.840	1.41E-87
*LINC01116*	*OLFML2B*	0.810	0.770–0.840	5.51E-87
*LINC01116*	*NTNG1*	0.810	0.770–0.840	5.81E-87
*LINC01116*	*SLC17A7*	0.810	0.770–0.840	9.88E-87
*LINC01116*	*NTRK1*	0.800	0.760–0.840	1.44E-84
*LINC01116*	*MRC2*	0.800	0.760–0.830	1.35E-83
*LINC01116*	*SLC7A3*	0.800	0.760–0.830	1.69E-83

*LncRNA* long non-coding RNA, *PCG* protein-coding gene, *CI* confidence interval

### Analysis of differential expressions and diagnostic potential

**Fig. 2:**
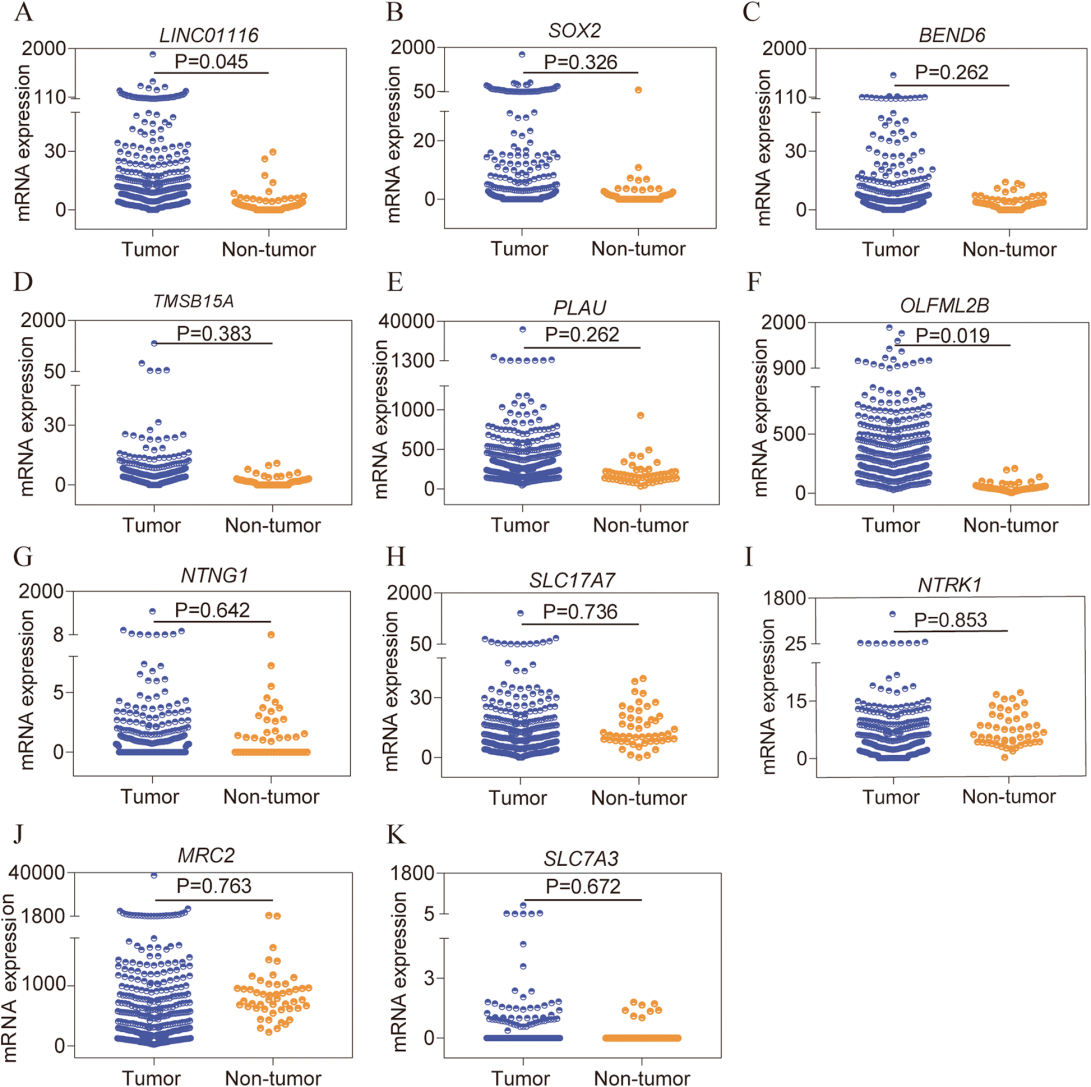
Scatter plots of LINC01116 and ten protein-coding genes in HCC. **A**-**K**: Scatter plots of LINC01116, *SOX2*, *BEND6*, *TMSB15A*, *PLAU*, *OLFML2B*, *NTNG1*, *SLC17A7*, *NTRK1*, *MRC2*, and *SLC17A3* in HCC, respectively

**Fig. 3:**
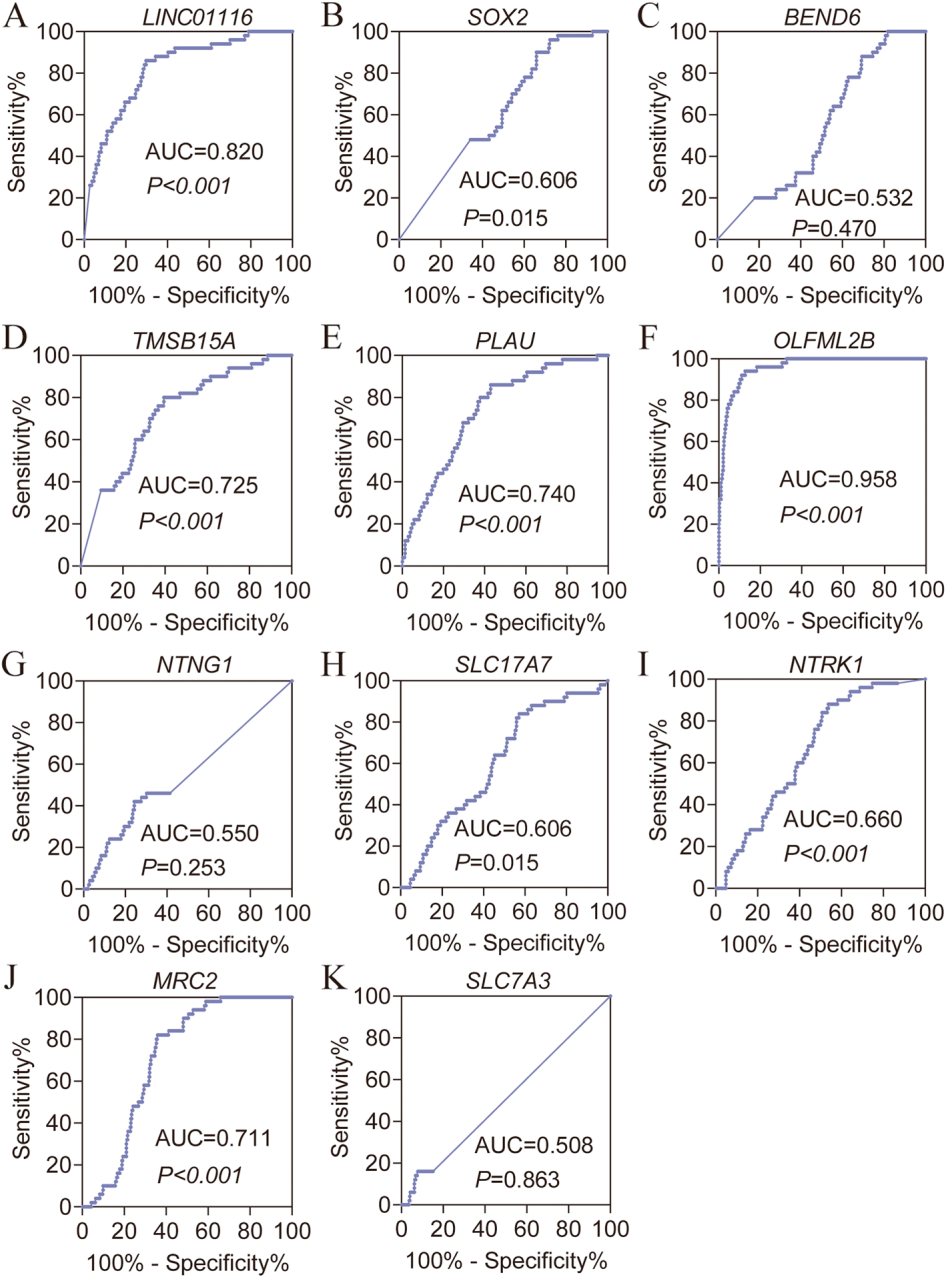
Diagnostic ROC curves of LINC01116 and ten protein-coding genes in HCC. **A**-**K** Diagnostic ROC curves of LINC01116, *SOX2*, *BEND6*, *TMSB15A*, *PLAU*, *OLFML2B*, *NTNG1*, *SLC17A7*, *NTRK1*, *MRC2*, and *SLC17A3* in HCC, respectively

Differential expressions analysis using the MERAV database indicated that LINC01116, *BEND6*, *PLAU*, *OLFML2B*, *SLC17A7*, and *SLC7A3* were significantly different from LINC01116 (Figure S1 A, C, E, F, H, K), while the others were not. Differential expression analysis showed that LINC01116 and *OLFML2B* were differentially expressed, with higher expression in tumor tissues (*P* = 0.045, 0.019, Fig. 2A, F). However, other biomarkers did not show statistical significance (*P* > 0.05, Fig. 2B-E, G-K). In terms of the diagnostic potentials of the various genes, only LINC01116, *TMSB15A*, *PLAU*, *OLFML2B,* and *MRC2* were found to have the potential of aiding in HCC diagnosis (all AUC ≥ 0.700, all *P* ≤ 0.05, Fig. 3A, D-F, J, Table S2) but had not in other biomarkers (all AUC < 0.700, Fig. 3B, C, G, H, I, K).

### Survival analysis and conjoint analysis

**Fig. 4:**
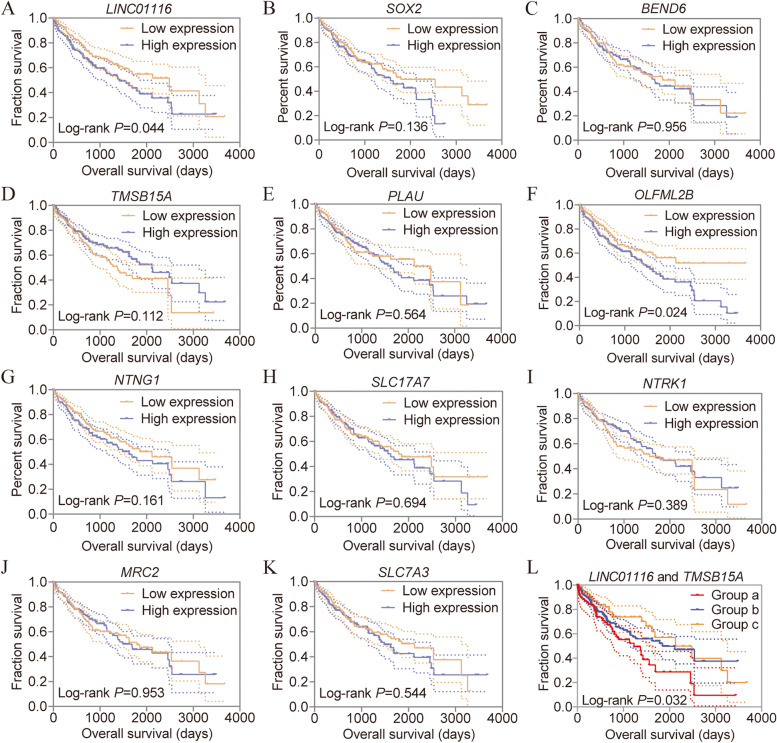
Survival and conjoint analyses of LINC01116 and ten protein-coding genes in HCC. **A**-**K** Survival analysis of LINC01116, *SOX2*, *BEND6*, *TMSB15A*, *PLAU*, *OLFML2B*, *NTNG1*, *SLC17A7*, *NTRK1*, *MRC2*, and *SLC17A3* in HCC, respectively; **L** Conjoint survival analysis of LINC01116 and TMSB15A in HCC

Survival analysis of LINC01116 and PCGs were performed using the univariate Cox hazard regression model. The model showed that LINC01116 and *OLFML2B* have prognostic significance (crude *P* = 0.044, 0.024, respectively. Table 2, Fig. 4A, F) but had not in other biomarkers (all AUC < 0.700, Fig. 4B-E, G-K). We then conducted a multivariate cox regression model using prognosis-related clinical factors and these genes. Multivariate analysis revealed that LINC01116 and *TMSB15A* have prognostic significance (adjusted *P* = 0.046, 0.003, respectively, Table 2). Further, conjoint analysis for LINC01116 and *TMSB15A* was performed and showed distinguished survival among groups a, b, and c (crude *P* = 0.032, adjusted *P* = 0.002, Table 3, Fig. 4L).

**Table 2:** Survival analysis of *LINC01116* and target genes in hepatocellular carcinoma

Variables	Patients	Overall survival					
	(*n* = 370)	No. of event	MST (days)	HR (95%CI)	Crude *P* value	HR (95%CI)	Adjusted *P* value^§^
*LINC01116*							
Low expression	185	55	2486	Ref.	**0.044**	Ref.	**0.046**
High expression	185	75	1423	**1.430 (1.009–2.027)**		**1.493 (1.007–2.214)**	
*SOX2*							
Low expression	185	63	1791	Ref.	0.136	Ref.	0.124
High expression	185	67	1560	1.305 (0.920–1.850)		1.365 (0.918–2.028)	
*BEND6*							
Low expression	185	63	1852	Ref.	0.956	Ref.	0.416
High expression	185	67	1685	1.010 (0.715–1.426)		0.852 (0.580–1.253)	
*TMSB15A*							
Low expression	185	69	1372	Ref.	0.112	Ref.	**0.003**
High expression	185	61	2116	0.754 (0.532–1.068)		**0.540 (0.362–0.806)**	
*PLAU*							
Low expression	185	57	2131	Ref.	0.564	Ref.	0.560
High expression	185	73	1624	1.108 (0.782–1.570)		0.892 (0.608–1.309)	
*OLFML2B*							
Low expression	185	51	NA	Ref.	**0.024**	Ref.	0.520
High expression	185	79	1423	**1.503 (1.056–2.139)**		1.139 (0.767–1.691)	
*NTNG1*							
Low expression	185	60	2116	Ref	0.161	Ref.	0.644
High expression	185	70	1624	1.280 (0.906–1.809)		1.096 (0.742–1.621)	
*SLC17A7*							
Low expression	185	64	1791	Ref.	0.694	Ref.	0.828
High expression	185	66	1622	1.072 (0.759–1.512)		0.958 (0.650–1.411)	
*NTRK1*							
Low expression	185	66	1560	Ref.	0.389	Ref.	0.053
High expression	185	64	1694	0.859 (0.608–1.214)		0.681 (0.462–1.004)	
*MRC2*							
Low expression	185	65	1791	Ref.	0.953	Ref.	0.280
High expression	185	65	1685	0.990 (0.701–1.397)		0.805 (0.543–1.193)	
*SLC7A3*							
Low expression	185	64	2131	Ref.	0.544	Ref.	0.749
High expression	185	66	1490	1.113 (0.788–1.571)		0.938 (0.633–1.390)	
Risk score model							
Low risk	185	57	2116	Ref.	**0.030**	Ref.	**0.018**
High risk	185	73	1386	**1.469 (1.038–2.078)**		**1.595 (1.082–2.351)**	

*Abbreviations: NA* Not available, *MST* Median survival time, *HR* hazard ratio, *95%CI* 95% confidence interval

^**§**^: *P* values were adjusted for radical resection, tumor stage and HBV infection; Bold indicates significant *P* values

**Table 3:** Joint-effect analysis of *LINC01116* and *TMSB15A* for overall survival

Group	*LINC01116*	*TMSB15A*	Overall survival					
	expression	expression	Events/total	MST (Days)	Crude HR (95%CI)	Crude *P* value	Adjusted HR (95%CI)	Adjusted *P* value^ɸ^
a	High	Low	40/87	1229	Ref.	**0.032**	Ref.	**0.002**
b	High	High	64/196	1852	0.687 (0.462–1.021)	0.063	**0.591 (0.380–0.918)**	**0.019**
	Low	Low						
c	Low	High	26/87	2131	**0.526 (0.320–0.865)**	**0.011**	**0.365 (0.207–0.643)**	** < 0.001**

*Abbreviations: NA* Not available, *MST* Median survival time, *HR* Hazard ratio, *95%CI* 95% confidence interval

ɸ: *P* values were adjusted for radical resection, tumor stage and HBV infection; Bold indicates significant *P* values

### Construction of predictive model using risk scores and nomogram

**Fig. 5:**
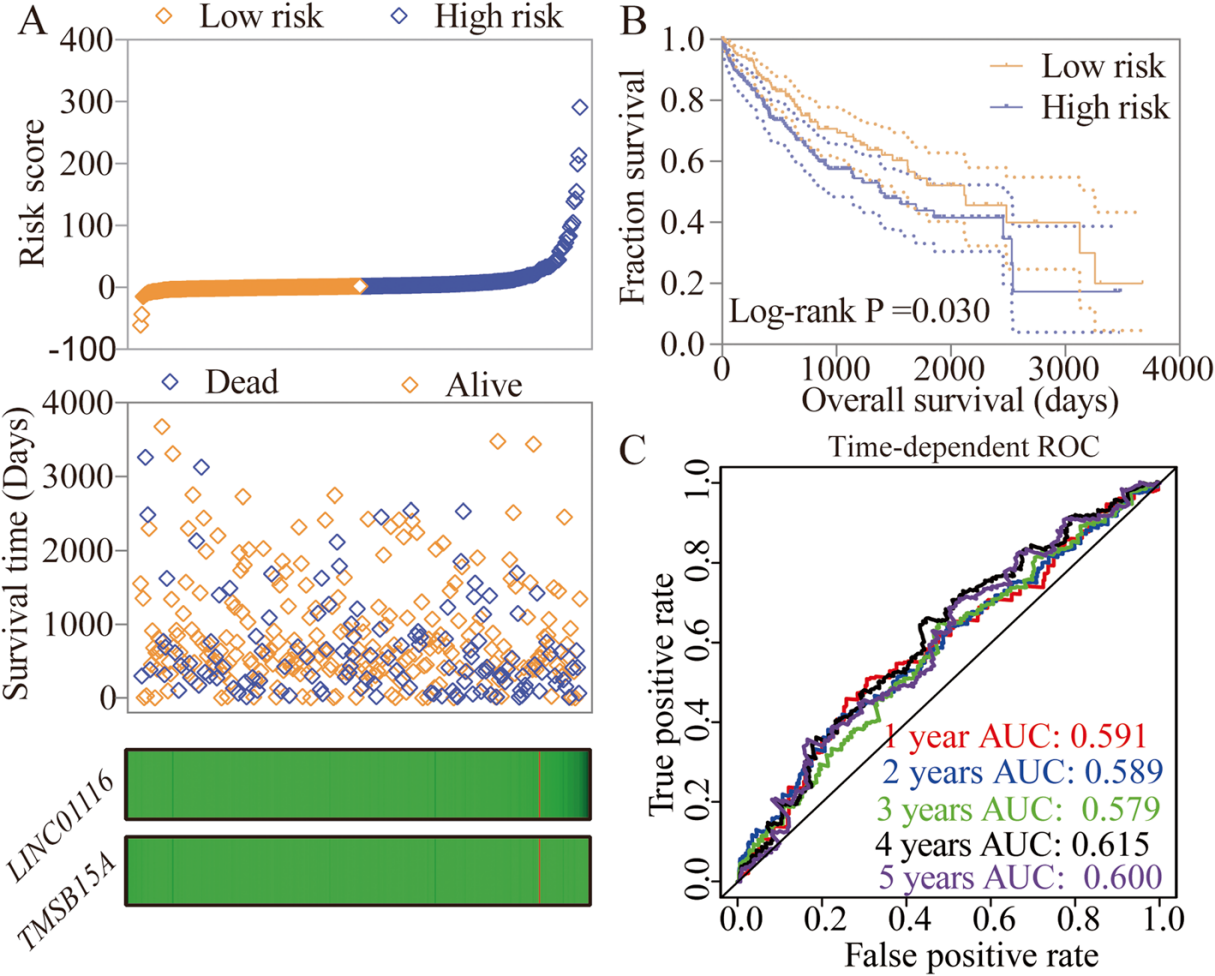
Risk score model, survival plot, and ROC curves of LINC01116 and *TMSB15A*. **A** Risk score model including risk scores, survival, and heatmaps; **B** Survival plot of risk scores by median cutoff; **C** Time-dependent ROC curves of risk scores at 1–5 years

**Fig. 6:**
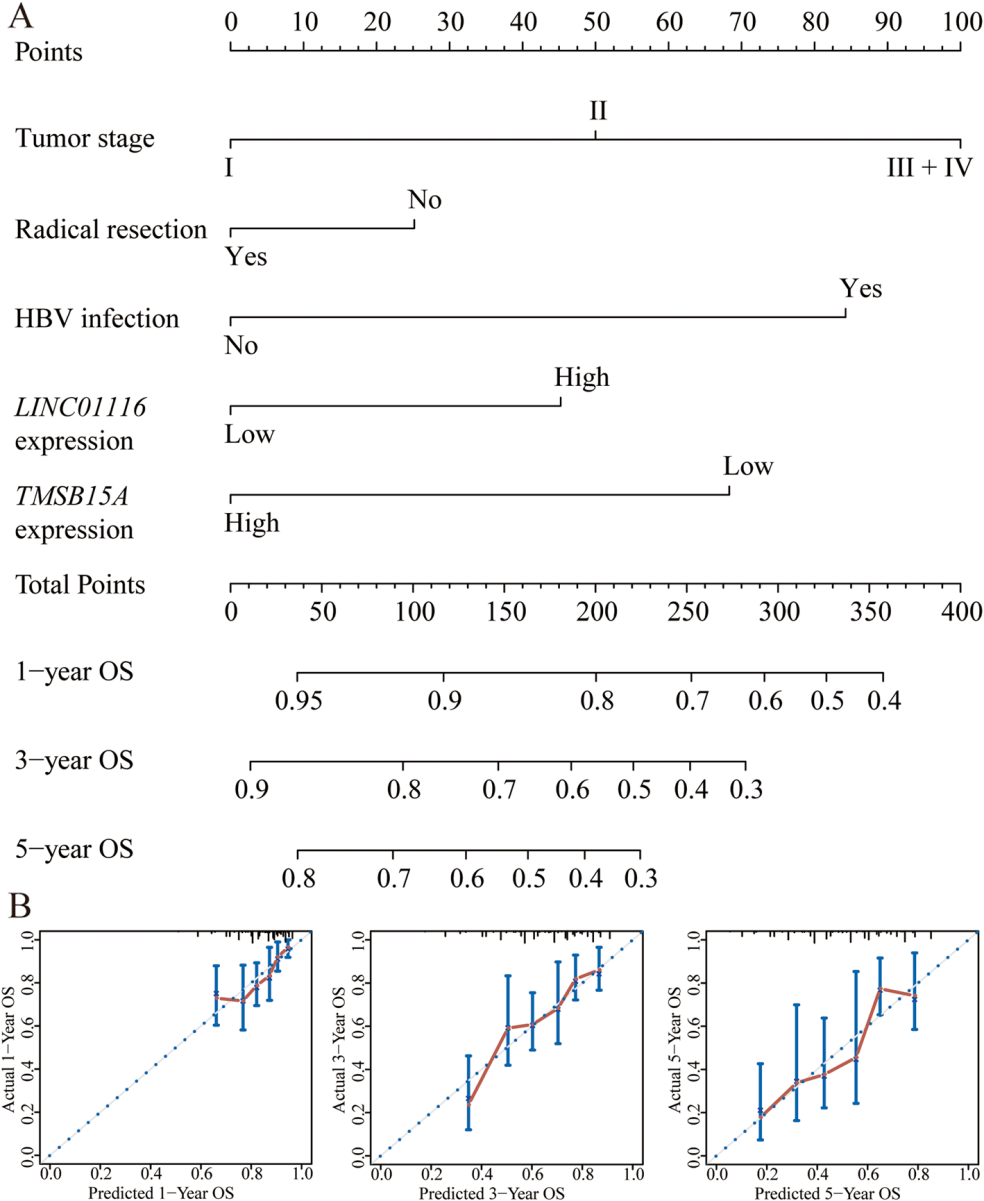
Nomogram of clinical factors, LINC01116 and TMSB15A, and calibration plots. **A** Nomogram of tumor stage, radical resection, HBV infection status, LINC01116, and TMSB15A expressions to predict 1, 3-, and 5-year survival probability. **B** Calibration plots at 1, 3-, and 5-year of the nomogram

A risk score model was constructed using LINC01116, *TMSB15A* expressions, and HBV status, and tumor stage and radical resection via a multivariate cox hazard model (Fig. 5A, Table 4). The identified elements of risk score include risk score rank, survival status, and heatmap of the expression of LINC01116 and *TMSB15A*. Risk scores were divided into low and high-risk groups at median cutoff. A Kaplan–Meier plot was drawn using low and high-risk groups (crude *P* = 0.030, Fig. 5B, Table 2). After that, time-dependent ROC curves were drawn at 1–5 year, which revealed similar prediction results (Fig. 5C). A nomogram was constructed using the expressions of LINC01116, TMSB15A and HBV status, and tumor stage and radical resection based on the different points of each factor (Fig. 6). Tumor stage I, radical resection, without HBV infection, low expression of LINC01116, and high expression of *TMSB15A* indicated lower points, which therefore suggested a better OS prediction at 1, 3-, and 5-years (Fig. 6A). Internal validations were conducted using C-index for predicted and actual OS status (Fig. 6B).

**Table 4:** Risk score model constructed by *LINC01116* and *TMSB15A*

Variables	β	SE	Wald	*P* value	HR (95% CI)
Tumor stage I			15.251	< 0.001	
Stage II	0.378	0.267	1.995	0.158	1.459 (0.864–2.463)
Stage III + IV	0.896	0.231	15.099	0.000	2.450 (1.559–3.849)
Radical resection	0.215	0.356	0.364	0.547	1.240 (0.617–2.492)
HBV infection	-0.760	0.262	8.397	0.004	0.468 (0.280–0.782)
*LINC01116*	0.401	0.201	3.963	0.047	1.493 (1.006–2.215)
*TMSB15A*	-0.615	0.204	9.081	0.003	0.541 (0.362–0.806)

### Exploration of molecular mechanisms via GSEA

**Fig. 7:**
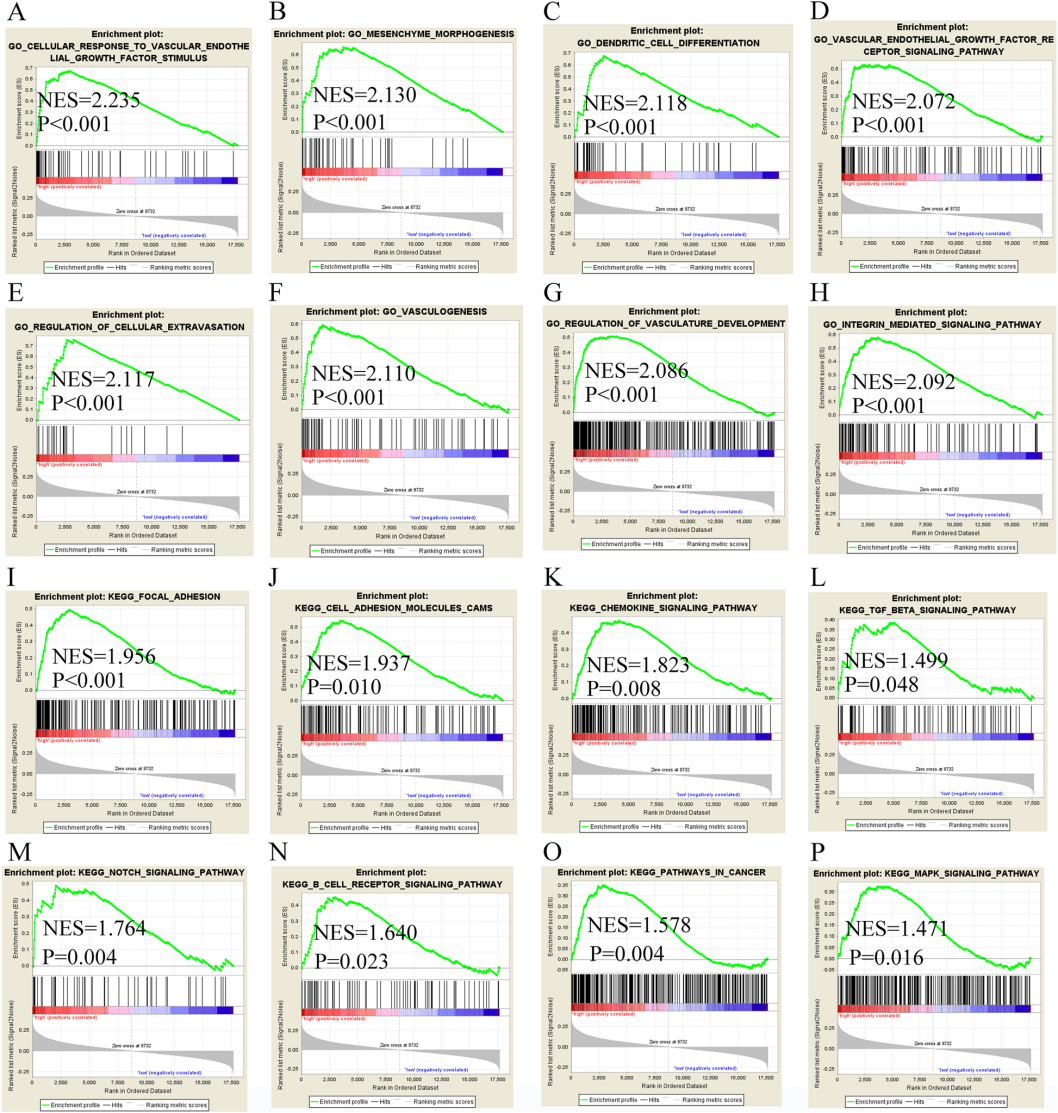
Results showing the molecular mechanisms of LINC01116 may be involved in HCC. **A**-**H** Gene ontology terms of LINC01116 may be involved in HCC; **I**-**P** KEGG pathways of LINC01116 may be involved in HCC

We explored the potential molecular mechanisms of LINC01116 and *TMSB15A* that could be involved in HCC prognosis. We then analyzed gene ontology (GO) terms and the Kyoto encyclopedia of genes and genomes (KEGG) pathways to identify the specific mechanisms. Specifically, LINC01116 enriched in several GO terms, including cellular response to vascular endothelial growth factors stimulus, mesenchymal morphogenesis, dendritic cell differentiation, vascular endothelial growth factor receptor signaling pathway, vasculogenesis, and integrin-mediated signaling pathway (Fig. 7A-H). The enriched KEGG pathways participate in focal adhesion, cell adhesion molecular cams, chemokine signaling, TGF-β signaling, notch signaling, B cell receptor signaling, pathways in cancer, and MAPK signaling (Fig. 7I-P). *TMSB15A* was enriched in GO terms involved in negative regulation of endothelial cell proliferation, blood vessel endothelial cell migration, stem cell division, mesenchyme development, and vasculogenesis (Figure S2 A-H). *TMSB15A* was enriched in KEGG pathways involved in drug metabolism, other enzymes, peroxisome, propanoate metabolism, and steroid hormone biosynthesis (Figure S2 I-L).

### Identification of candidate target drugs and interaction networks of LINC01116

**Fig. 8:**
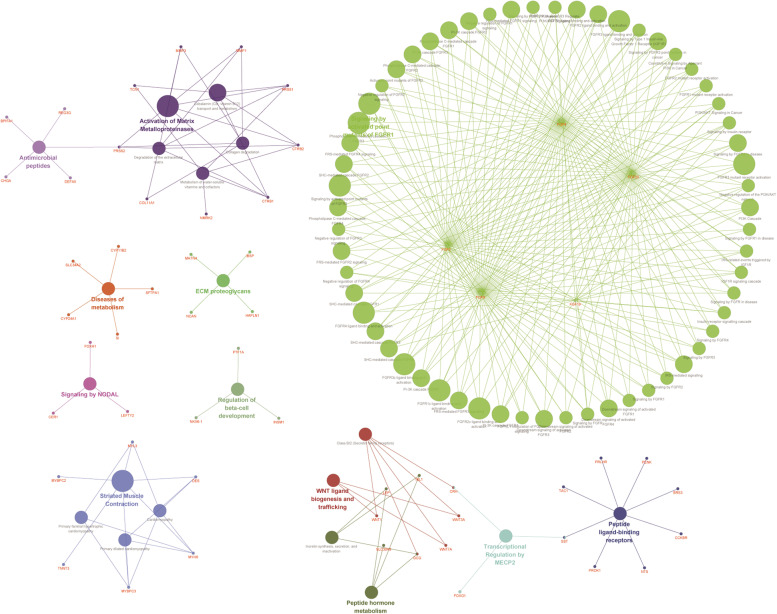
Metabolic pathways, diseases, and gene ontology terms of differentially expressed genes dependent on the expression of LINC01116

**Fig. 9:**
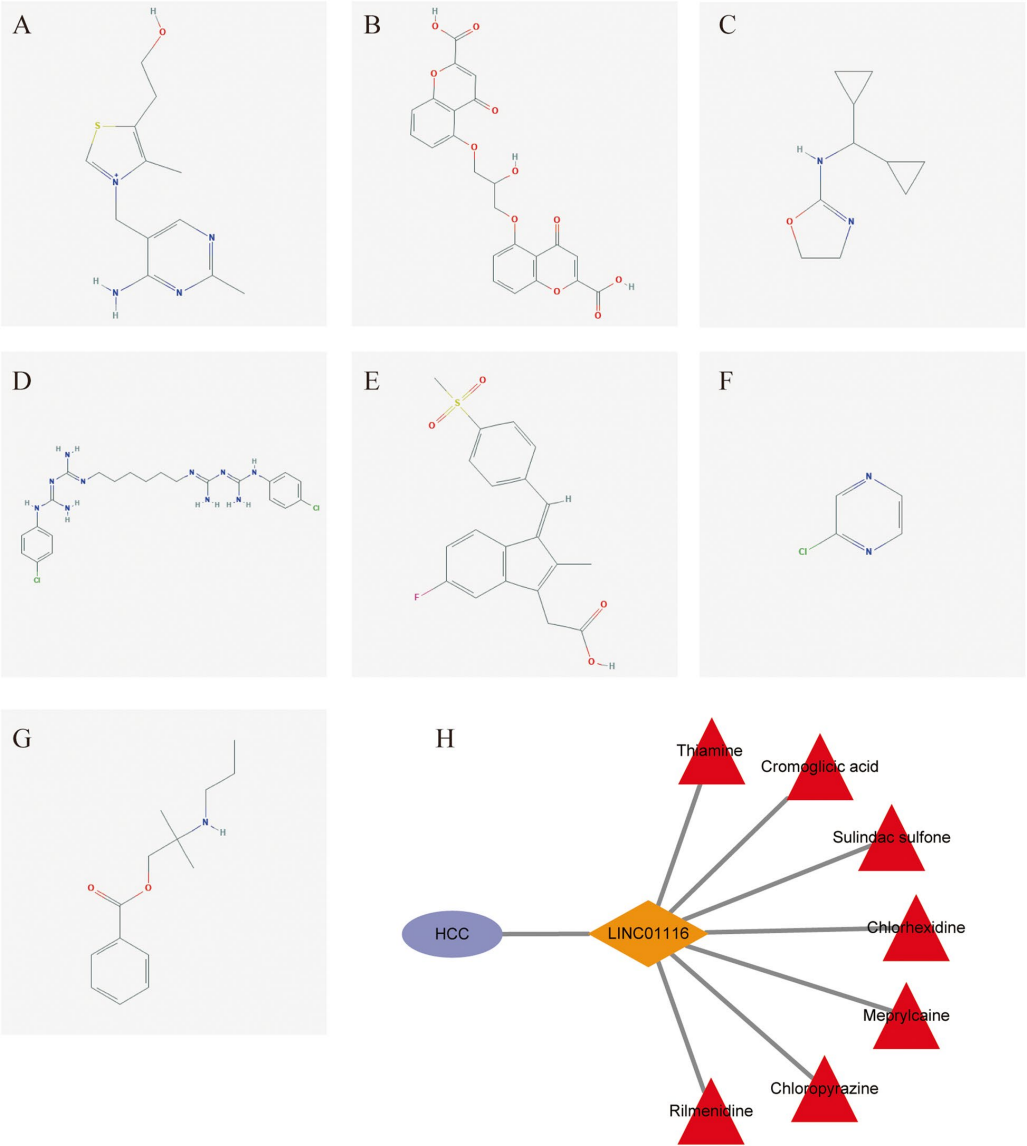
Potential drugs that target LINC01116 and their respective interaction plot in HCC. **A**-**G** Thiamine, Cromolyn, Rilmenidine, Chlorhexidine, Sulindac_sulfone, Chloropyrazine, Meprylcaine aimed at; **H** Interaction plot among target drugs, LINC01116, and HCC

Using |fold change|≥ 2 and *P* ≤ 0.05, we identified a total of 171 up-regulated and 37 down-regulated genes. We then used these DEGs to construct interaction networks, including KEGG pathways and diseases (Fig. 8). This interactive network was associated with metabolic diseases, peptide hormone metabolism, NODAL signaling, regulation of beta-cell development, WNT ligand biogenesis and trafficking, antimicrobial peptides, PI3K/AKT signaling in cancer, and signaling by the insulin receptor. After that, candidate target drugs were generated via the cMAP database using these DEGs and listed as follows: Thiamine, Cromolyn, Rilmenidine, Chlorhexidine, Sulindac_sulfone, Chloropyrazine, and Meprylcaine (Fig. 9, Table 5). Two dimensional (2D) structures of these drugs are shown in Fig. 9A-G. Our results show that the drugs have potential clinical significance, are negatively related to the expression of LINC01116, with its high expression indicating a poor outcome (Fig. 9H).

**Table 5:** Candidate pharmacological targets toward *LINC01116*

Name	PubChem CID	Mean	Enrichment	*P* value
Thiamine	1130	-0.783	-0.95	0.00022
Cromoglicic acid	2882	-0.831	-0.98	0.00087
Rilmenidine	68,712	-0.396	-0.845	0.00103
Chlorhexidine	9,552,079	-0.402	-0.671	0.00891
Sulindac sulfone	5,472,495	-0.555	-0.932	0.00936
Chloropyrazine	73,277	-0.394	-0.685	0.02158
Meprylcaine	4065	-0.391	-0.668	0.02741

### Immune infiltration and promoter methylation analysis of PCGs

**Fig. 10:**
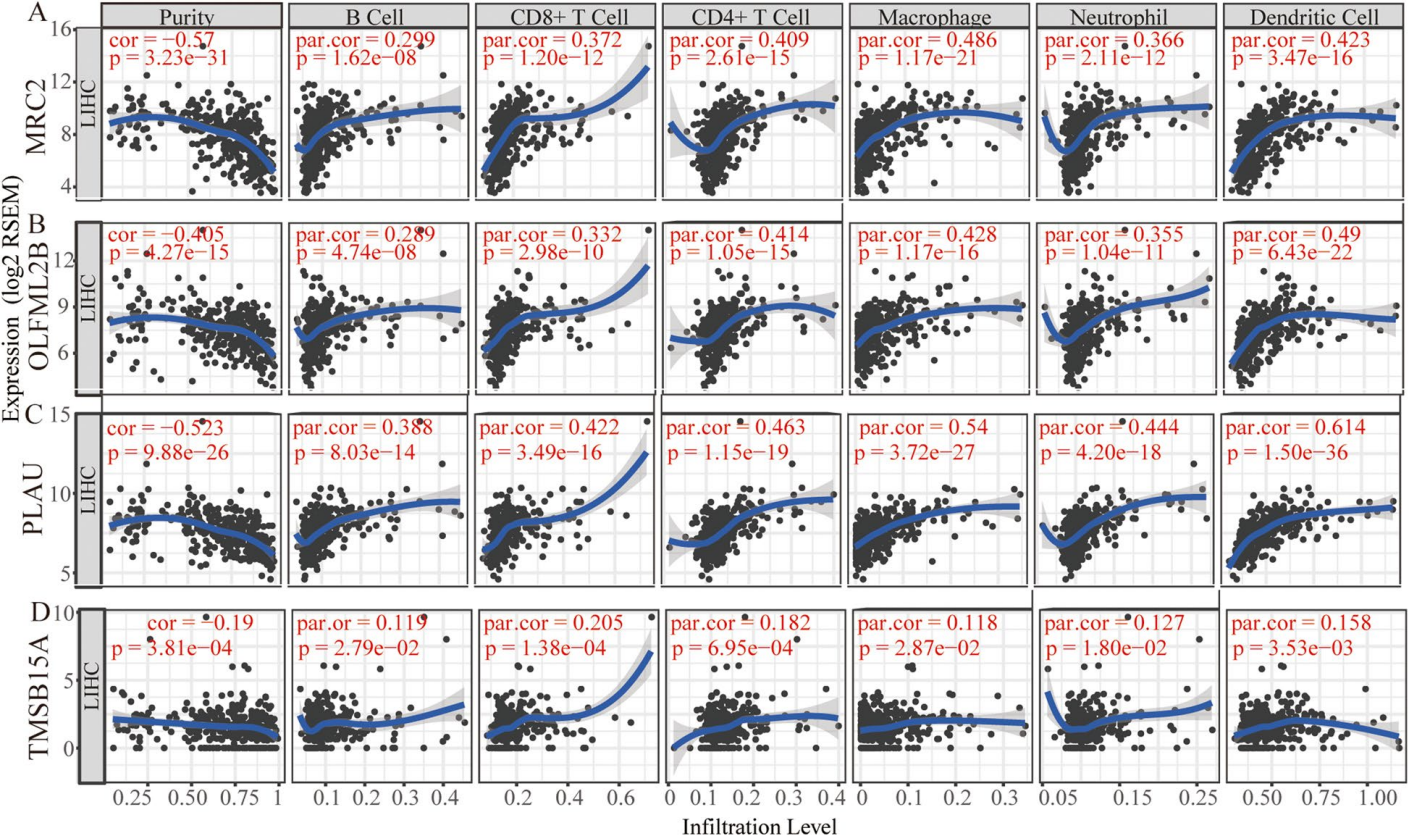
Analysis of immune infiltration between gene expressions and immune infiltrates, and purity. **A**-**D** Immune infiltration analysis between *MRC2*, *OLFML2B*, *PLAU,* and *TMSB15A* expressions and immune infiltrates, and purity, respectively

**Fig. 11:**
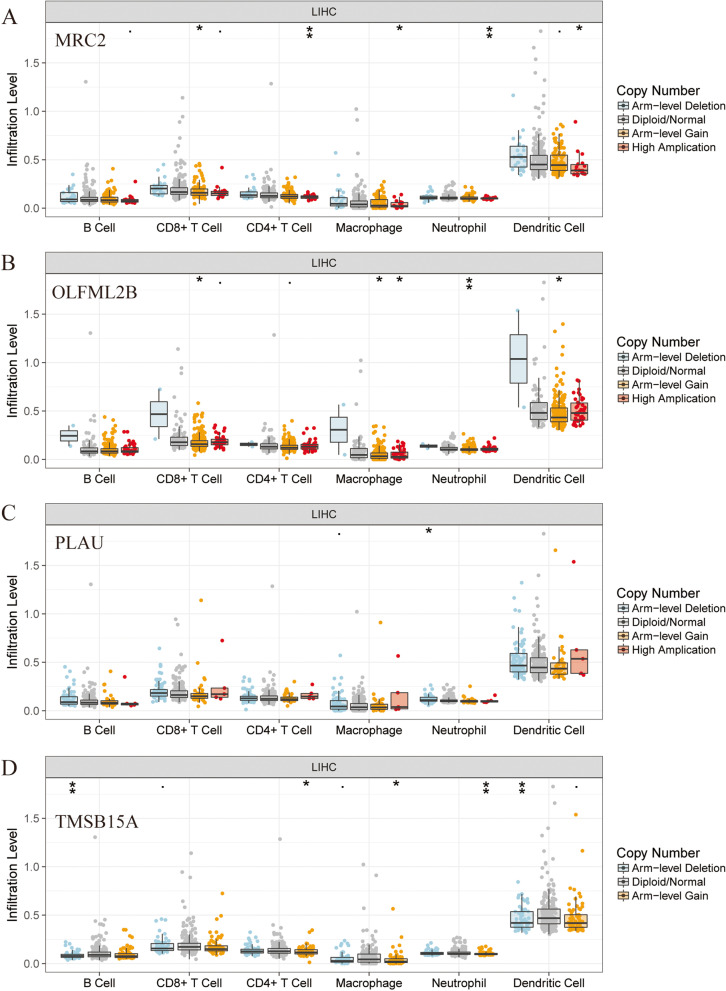
SCNA analysis of immune infiltrates using protein-coding genes. **A**-**D** SCNA analysis of immune infiltrates in *MRC2*, *OLFML2B*, *PLAU,* and *TMSB15A*, respectively

Due to the unavailability of LINC01116 in TIMER and UALCAN, only PCGs were conducted in the analysis of immune infiltration and methylation. The analysis of immune infiltration revealed that all the four PCGs (*MRC2*, *OLFML2B*, *PLAU*, *TMSB15A*) were negatively associated with the purity (all *P* < 0.001, *r* < 0, Fig. 10). Meanwhile, all the four PCGs were positively associated with specific cell types, including B cell, CD8^+^ T cell, CD4^+^ T cell, macrophage, neutrophil, and dendritic cells (all *P* < 0.050, *r* > 0). Then, SCNA analysis indicated that all of the four genes were partially associated with SCNA among B cell, CD8^+^ T cell, CD4^+^ T cell, macrophage, neutrophil, and dendritic cells (Fig. 11). Specifically, *MRC2* and *OLFML2B* showed significance in arm-level gain and high amplification; PLAU showed significance in arm-level deletion, while *TMSB15A* exhibited significance in arm-level deletion and gain.

**Fig. 12:**
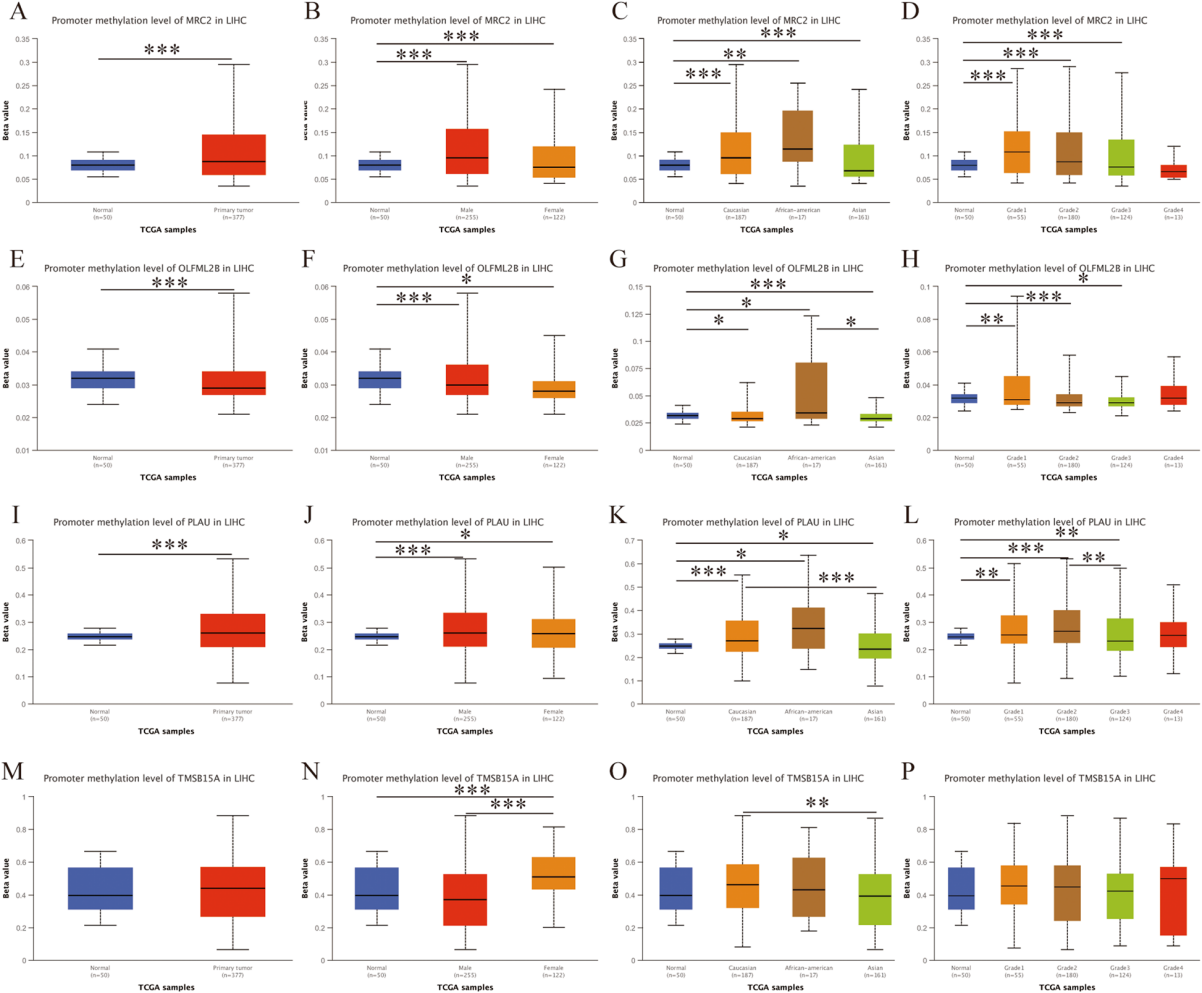
Differential analysis of promoter methylation of protein-coding genes in HCC. **A**-**D** Differential analysis of promoter methylation of *MRC2* by tumor, gender, race and tumor grade; **E**–**H** Differential analysis of promoter methylation of *OLFML2B* by tumor, gender, race and tumor grade; **I**-**L** Differential analysis of promoter methylation of *PLAU* by tumor, gender, race and tumor grade; **M**-**P** Differential analysis of promoter methylation of *TMSB15A* by tumor, gender, race and tumor grade

Analysis of promoter methylation demonstrated that *MRC2*, *OLFML2B*, and *PLAU* revealed differential and high methylation levels in primary tumors compared with normal (all *P* < 0.001, Fig. 12A, E, I). However, no significant differences were observed in *TMSB15A* (Fig. 12M). Methylation analysis by gender demonstrated that *MRC2*, *OLFML2B*, and *PLAU* have differential and high methylation in HCC tissues of both male and female populations compared with healthy tissues (all *P* < 0.050, Fig. 12B, F, J). However, *TMSB15A* showed differential methylation between males and females (Fig. 12N). Methylation analysis by race suggested that *MRC2*, *OLFML2B*, and *PLAU* have differential significance between normal and other races, including Caucasian, African-American, and Asian (Fig. 12C, G, K). However, TMSB15A showed a difference between the Caucasian and Asian populations (Fig. 12O). Methylation analysis by tumor grade suggested that *MRC2*, *OLFML2B*, and *PLAU* have differential significance between normal and tumor grades 1–3 (Fig. 12D, H, L) while TMSB15A showed no difference between normal tissues and tumor grade (Fig. 12P).

### Construction of ceRNA network and validations of clinical significance by oncomine database

**Fig. 13:**
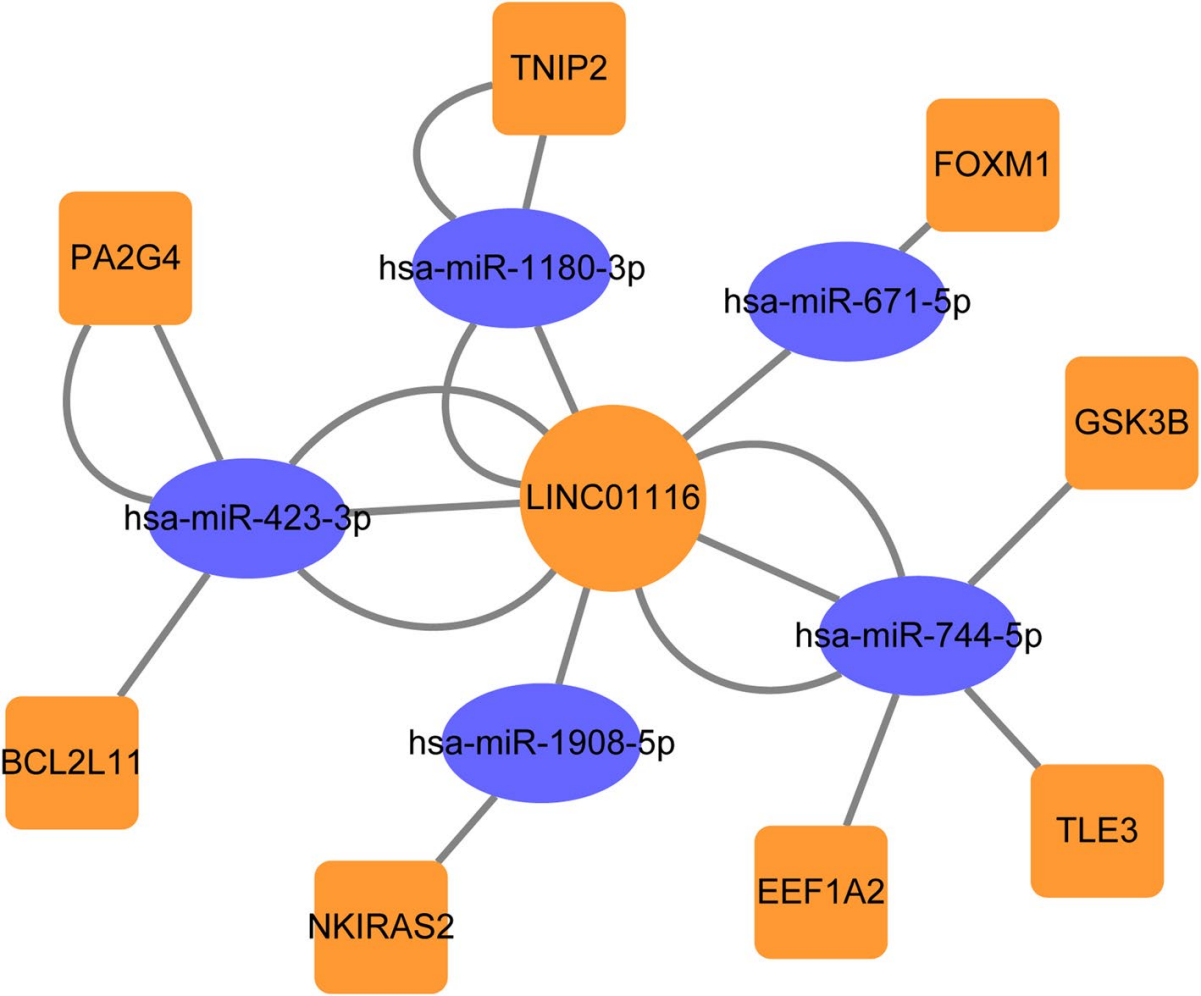
Construction of ceRNA network containing mRNA, miRNA, and lncRNA of LINC01116-related

A ceRNA network was constructed based on negative regulation relationship with LINC01116 (Fig. 13). Specifically, LINC01116 was connected with miR-423-3P, miR-1908-5P, miR-744-5P, miR-1180-3P, miR-671-5P, *GSK3B*, *FOXM1*, *TNIP2*, *PA2G4*, *BCL2L11*, *NKIRAS2*, *EEF1A2*, *TLE3*. Then, validation by the Oncomine database suggested that *OLFML2B*, *PLAU*, and *MRC2* have differential expressions and diagnostic potentials for HCC in the two datasets (all *P* < 0.050, all AUC > 0.700, Figure S3 C-H, K-P). However, *TMSB15A* showed diagnostic potentials in only one dataset (Figure S3 A-B, I-J).

## Discussion

Application of high-throughput sequencing technology and bioinformatics methodologies have led to the discovery that PCGs consist of approximately 2% of the entire human genome. The rest of the human genome comprises thousands of non-coding RNAs, including LncRNAs [28, 29]. Recent evidence suggest that LincRNAs play crucial roles in the pathogenesis of multiple tumors. They do so by influencing several cellular functions, such as cell proliferation, differentiation, metastasis, and drug resistance [30, 31].

New discoveries involving lincRNAs have advanced our understanding on the initiation and progression of cancers. A study by Panzitt et al. revealed a novel mRNA-like to be the most up-regulated gene in HCC [32]. Matok et al. found that HULC is up-regulated in colorectal carcinoma thereby accelerating metastasis of colorectal carcinoma cells to liver tissues indicating that HULC has an important role in HCC [33]. Elsewhere, it was reported that HOTAIR associates with polycomb repressive complex 2, trimethylate H3K27 to repress the transcription levels of metastasis-related gene suppressors, therefore increase the invasiveness and metastasis of breast cancer [34, 35]. Overexpression of HOTAIR in HCC tissues preducted high recurrence [36]. The same study showed that knockdown of HOTAIR decreased the invasiveness and viability of HepG2 cells [36]. Inversely, HepG2 cell line was led to a significant increase after the apoptotic stimuli TNF-α as well as chemotherapeutic drug cisplatin and doxorubicin [36].

Given the aforementioned evidence that lincRNAs participate in other cancers, we explored their roles in HCC. LINC01116 regulates diverse cancers, including glioma, HNSCC, breast cancer, osteosarcoma, epithelial ovarian cancer and oral squamous cell carcinoma. For instance, it was found that LINC01116 was not only significantly highly expressed in glioma tissues but also associated with an increased risk of recurrence and poor OS [15]. The same study showed that LINC01116 regulates tumorigenesis of gliomas by targeting VEGF and modulating expression of VEGF by competitive adsorption of micorRNA-31-5p at the posttranscriptional level [15]. Further analysis confirmed that LINC01116 may serve as a valuable auxiliary prognostic biomarker and prognostic indicator for glioma patients [15]. In oral squamous cell carcinoma and nasopharyngeal carcinoma tissues, LINC01116 is not only up-regulated but also may serve as a valuable diagnostic biomarker and can predict the prognosis of HNSCC [16]. Consistently, our study reveals that LINC01116 may serve as a diagnostic biomarker and prognostic indicator for HCC patients. Previously, Hao et al. reported that LINC01116, DUXAP8, LINC01138 and PCAT6 were dysregulated and significantly associated with poor prognosis of HCC [37].

In this study, GSEA analysis revealed that LINC01116 may regulate cellular responses to VEGF stimuli, VEGF receptor signailing pathway, mesenchyme morphogenesis, focal adhesion, cell adhesion molecular cams, chemokine signaling pathway, TGF β signaling pathway, notch signaling pathway, dendritic cell differentiation, and B cell receptor signaling pathway in cancer. These findings on VEGF stimuli, immune infiltration results, and VEGF receptor signailing pathway are parallel with those reported by Jingliang et al. [15, 16].

Studies have shown that lincRNAs regulate various cellular processes, such as cell cycle, immune surveillance and stem cell pluripotency [38, 39]. Molecular mechanistic tests by GSEA analysis demonstrated that *TMSB15A* is enriched in stem cell division, mesenchyme development, vasculogenesis while LINC01116 modulates the differentiation of dendritic cells and B cell receptor signaling pathway. These results are consistent with of the aforementioned studies in stem cell and immune surveillance aspects. Further, immune infiltration analyses suggested that four PCGs (*MRC2*, *OLFML2B*, *PLAU*, and *TMSB15A)* were negatively associated with the purity while the four PCGs were positively associated with specific cell types, including B cell, CD8^+^ T cell, CD4^+^ T cell, macrophage, neutrophil and dendritic cell. These results are congruent with those mentioned previously in the above studies. *MRC1* and *MRC2* are crucial components of the innate immune system, and they contribute to defense against pathogenic bacterial infections [40]. Moreover, they are highly expressed in liver tissues compared to spleen and kidney in challenged fish. *MRC2* participates in lysosomal collagen degradation [41] and is required for T_reg_ differentiation in the ectopic lesion, especially for CD4^high^ T_reg_ [42].

*OLFML2B*, highly expressed in ovary, was found to be the most destructive single nucleotide polymorphisn (up to 61) compared with *OLFM2*, *OLFM4* and *LPHN2*, without mutations [43]. It was, therefore, inferred that Olfactomedin protein modulates the immune function and development in the nerve system [43]. Similarly, our study reveals that this protein plays an important role in the immune system. Immune-related signature *PLAU* was found differentially and highly expressed in esophageal squamous cell cancer and involved in the general immune response [44]. PLAU was also found to predict poor prognosis of esophageal squamous cell carcinoma [44]. In contrast, it is not know whether *TMSB15A* is related to immune functions. In this study, SCNA analysis indicated that four genes were partially associated with B cells, CD8^+^ T cells, CD4^+^ T cells, macrophages, neutrophils and dendritic cells. Specifically, *MRC2* and *OLFML2B* showed apparent arm-level gain and high amplification; PLAU showed apparent arm-level deletion while *TMSB15A* showed significant arm-level deletion and gain. However, the association between these genes and SCNA has not been documented.

In addition, this study reveals that these four genes possess diagnostic potential for HCC. It was previously reported that *OLFML2B* was overexpressed in gastric cancer tissues compared to normal gastric tissues and exhibited moderate diagnostic potential (AUC = 0.867, *P* < 0.0001) and was associated with poor survival of gastric cancer [45]. Similarly, our TCGA, Oncomine database findings reveal that *OLFML2B* is differentially and highly expressed, with diagnostic value in HCC. However, in our study, *OLFML2B* was not found to possess prognostic significance in HCC. Xiaohong et al. reported that *MRC2* predicted poor prognosis of HCC by regulating TCGβ1 [46] but the study did not explore its diagnostic potential. No study has documented the diagnostic value of *MRC2*. For PLAU, it was found to be aberrantly expressed in HNSCC and that it can be used for diagnostic and prognostic purposes in HNSCC [47]. Specific, it was associated with invasiveness of HNSCC cells [47]. In this study, only its diagnostic potential and not prognostic significance was confirmed in HCC. Further investigations are advocated to confirm the prognostic value of *MRC2* and *PLAU* in HCC.

The potential target drugs of LINC01116 were as follows: Thiamine, Cromolyn, Rilmenidine, Chlorhexidine, Sulindac_sulfone, Chloropyrazine, and Meprylcaine. Thiamine was to act on metabolic pathways such as glycosaminoglycan degradation in a pilot study on type 2 diabetes mellitus-related HCC [48]. The study concluded that diabetes mellitus may influence the occurrence and progression of HCC by modulating various metabolic and immunity processes [48]. Thiamine compromised the anticancer efficacy of methrotrexate by ameliorating diethyl nitrosamine-induced HCC in wistar strain rats [49]. Sulindac_sulfone inhibited colon cancers in a k-ras (codon 12) mutation-independent manner [50]. Chlorhexidine was exhibited superior anti-tumor properties than cranberry extract in oral cancer AW13516 and KB cell lines [51]. Pyrazine diazohydroxide, was found to be a novel antineoplastic agent in a phase I and pharmacokinetic study [52]. The clinical value of drugs targeting LINC01116 in liver cancer should be investigated further.

DNA methylation modulates cell differentiation and is involved in tumorigenesis [53]. Previous evidence indicates that epigenetic markers can be used for prognostic and diagnostic purposes in oncology [54]. Promoter methylation analysis demonstrated that *MRC2*, *OLFML2B*, and *PLAU* were differentially and highly methylated in primary tumor cells compared with normal cells. Moreover, *MRC2*, *OLFML2B*, and *PLAU* were differentially and highly methylated between tumor and normal tissues as well as between genders, races and tumor grads. However, the diagnostic and prognostic significance of these genes in HCC need to be further investigated.

In addition, since the review process of this manuscript, Haisu Tao et al. had reported LINC01116 functioning as an immune and epithelial mesenchymal transition-related oncogene in HCC [55]. And their experiment indicated that LINC01116 promotes cell proliferation, cell cycle progression and tumor metastasis. To sum, our study found that LINC01116, *TMSB15A*, *PLAU*, *OLFML2B,* and *MRC2* have diagnostic potentials while LINC01116 and *TMSB15A* have prognostic significance in HCC. LINC01116 was enriched in the vascular endothelial growth factor (VEGF) receptor signaling pathway, mesenchyme morphogenesis, etc. Candidate drugs analysis identified: Thiamine, Cromolyn, Rilmenidine, Chlorhexidine, Sulindac_sulfone, Chloropyrazine, and Meprylcaine for therapeutic target. Then, immune infiltration revealed that *MRC2*, *OLFML2B*, *PLAU*, and *TMSB15A* are negatively associated with the purity but positively associated with the specific cell types. Analysis of promoter methylation demonstrated that *MRC2*, *OLFML2B*, and *PLAU* have differential and high methylation levels. Oncomine database identified the differential expressions and diagnostic potential of *OLFML2B.* Since our study demonstrated that LINC01116 has diagnostic significance and its association with the above ten biomarkers, further in vitro and in vivo functional studies could also be performed toward these aspects to further clarify its role in HCC.

This study has the following limitations. Our main findings need to be validated in other cohorts with more patients and clinical factors. In addition, in vivo and in vitro experiments should be performed to explore specific mechanisms of LINC01116 and PCGs in HCC. Thirdly, potential target drugs of LINC01116 for clinical application of HCC need future explores.

## Supplementary Information

**Additional file 1.**

## Data Availability

The datasets generated and/or analyzed during the current study are available in the TCGA repository (https://cancergenome.nih.gov/).
