# Supplementary material for: Comprehensive analysis of candidate signatures of long non-coding RNA LINC01116 and related protein-coding genes in patients with hepatocellular carcinoma
Source: BMC Gastroenterol. 2023 Jun 20;23:216. doi: 10.1186/s12876-023-02827-y (PMC10283182; doi:10.1186/s12876-023-02827-y)

| Table S1. Relationship between LINC01116 and mRNA in genome-wide | | | | | | |
| --- | --- | --- | --- | --- | --- | --- |
| gene1 | gene2 | t | df | cor | ci0.95 | p |
| LINC01116 | LINC01116 | Inf | 368 | 1 | 1, 1 | 0 |
| LINC01116 | SOX2 | 27 | 368 | 0.82 | 0.78, 0.85 | 1.87E-89 |
| LINC01116 | BEND6 | 27 | 368 | 0.81 | 0.78, 0.85 | 4.16E-89 |
| LINC01116 | TMSB15A | 26.7 | 368 | 0.81 | 0.77, 0.84 | 4.18E-88 |
| LINC01116 | PLAU | 26.6 | 368 | 0.81 | 0.77, 0.84 | 1.41E-87 |
| LINC01116 | OLFML2B | 26.4 | 368 | 0.81 | 0.77, 0.84 | 5.51E-87 |
| LINC01116 | NTNG1 | 26.4 | 368 | 0.81 | 0.77, 0.84 | 5.81E-87 |
| LINC01116 | SLC17A7 | 26.4 | 368 | 0.81 | 0.77, 0.84 | 9.88E-87 |
| LINC01116 | NTRK1 | 25.8 | 368 | 0.8 | 0.76, 0.84 | 1.44E-84 |
| LINC01116 | MRC2 | 25.6 | 368 | 0.8 | 0.76, 0.83 | 1.35E-83 |
| LINC01116 | SLC7A3 | 25.5 | 368 | 0.8 | 0.76, 0.83 | 1.69E-83 |
| LINC01116 | PCDH19 | 25.5 | 368 | 0.8 | 0.76, 0.83 | 3.45E-83 |
| LINC01116 | CRYBB1 | 25.4 | 368 | 0.8 | 0.76, 0.83 | 4.95E-83 |
| LINC01116 | COLEC12 | 25.4 | 368 | 0.8 | 0.76, 0.83 | 5.01E-83 |
| LINC01116 | DBX2 | 25.4 | 368 | 0.8 | 0.76, 0.83 | 5.83E-83 |
| LINC01116 | FABP7 | 25.4 | 368 | 0.8 | 0.76, 0.83 | 6.36E-83 |
| LINC01116 | KCNA1 | 25.4 | 368 | 0.8 | 0.76, 0.83 | 8.02E-83 |
| LINC01116 | HRH1 | 25.4 | 368 | 0.8 | 0.76, 0.83 | 8.44E-83 |
| LINC01116 | CRYBA4 | 25.4 | 368 | 0.8 | 0.76, 0.83 | 8.98E-83 |
| LINC01116 | PAX7 | 25.3 | 368 | 0.8 | 0.76, 0.83 | 1.03E-82 |
| LINC01116 | TNNT3 | 25.2 | 368 | 0.8 | 0.76, 0.83 | 3.59E-82 |
| LINC01116 | ATCAY | 25.2 | 368 | 0.8 | 0.75, 0.83 | 4.19E-82 |
| LINC01116 | IL31RA | 25.2 | 368 | 0.8 | 0.75, 0.83 | 4.87E-82 |
| LINC01116 | CRYGS | 25.1 | 368 | 0.79 | 0.75, 0.83 | 8.35E-82 |
| LINC01116 | CRYBA2 | 25.1 | 368 | 0.79 | 0.75, 0.83 | 1.28E-81 |
| LINC01116 | GPC1 | 25 | 368 | 0.79 | 0.75, 0.83 | 2.39E-81 |
| LINC01116 | PTN | 24.9 | 368 | 0.79 | 0.75, 0.83 | 6.92E-81 |
| LINC01116 | OSTN | 24.8 | 368 | 0.79 | 0.75, 0.83 | 1.14E-80 |
| LINC01116 | CHRNA1 | 24.8 | 368 | 0.79 | 0.75, 0.83 | 2.34E-80 |
| LINC01116 | SCUBE3 | 24.6 | 368 | 0.79 | 0.75, 0.82 | 6.52E-80 |
| LINC01116 | ALX4 | 24.5 | 368 | 0.79 | 0.75, 0.82 | 2.17E-79 |
| LINC01116 | CLEC11A | 24.5 | 368 | 0.79 | 0.74, 0.82 | 3.45E-79 |
| LINC01116 | HAPLN1 | 24.5 | 368 | 0.79 | 0.74, 0.82 | 3.53E-79 |
| LINC01116 | CHST2 | 24.4 | 368 | 0.79 | 0.74, 0.82 | 5.68E-79 |
| LINC01116 | STOML3 | 24.3 | 368 | 0.79 | 0.74, 0.82 | 1.33E-78 |
| LINC01116 | TLL2 | 24.3 | 368 | 0.78 | 0.74, 0.82 | 1.68E-78 |
| LINC01116 | AC024940.1 | 24.3 | 368 | 0.78 | 0.74, 0.82 | 1.95E-78 |
| LINC01116 | SRPX | 24.2 | 368 | 0.78 | 0.74, 0.82 | 6.86E-78 |
| LINC01116 | PCDH10 | 24.1 | 368 | 0.78 | 0.74, 0.82 | 8.50E-78 |
| LINC01116 | TMEM132B | 24.1 | 368 | 0.78 | 0.74, 0.82 | 8.69E-78 |
| LINC01116 | MSX1 | 24.1 | 368 | 0.78 | 0.74, 0.82 | 9.03E-78 |
| LINC01116 | MGAT5B | 24.1 | 368 | 0.78 | 0.74, 0.82 | 9.99E-78 |
| LINC01116 | ANO4 | 23.8 | 368 | 0.78 | 0.74, 0.82 | 1.29E-76 |
| LINC01116 | DRAXIN | 23.5 | 368 | 0.78 | 0.73, 0.81 | 2.29E-75 |
| LINC01116 | COL8A2 | 23.5 | 368 | 0.77 | 0.73, 0.81 | 2.80E-75 |
| LINC01116 | RPH3A | 23.1 | 368 | 0.77 | 0.72, 0.81 | 1.88E-73 |
| LINC01116 | ADD2 | 22.9 | 368 | 0.77 | 0.72, 0.81 | 5.68E-73 |
| LINC01116 | ABCA13 | 22.9 | 368 | 0.77 | 0.72, 0.81 | 1.11E-72 |
| LINC01116 | ADAMTS12 | 22.8 | 368 | 0.77 | 0.72, 0.8 | 1.86E-72 |
| LINC01116 | SLC35E4 | 22.8 | 368 | 0.76 | 0.72, 0.8 | 3.46E-72 |
| LINC01116 | RNF175 | 22.6 | 368 | 0.76 | 0.72, 0.8 | 1.66E-71 |
| LINC01116 | LCTL | 22.6 | 368 | 0.76 | 0.72, 0.8 | 2.43E-71 |
| LINC01116 | PDCD1LG2 | 22.5 | 368 | 0.76 | 0.72, 0.8 | 2.56E-71 |
| LINC01116 | EMID1 | 22.3 | 368 | 0.76 | 0.71, 0.8 | 1.69E-70 |
| LINC01116 | TUBA1A | 22.2 | 368 | 0.76 | 0.71, 0.8 | 4.62E-70 |
| LINC01116 | ADAMTS14 | 22 | 368 | 0.75 | 0.71, 0.79 | 3.89E-69 |
| LINC01116 | EFNB3 | 21.9 | 368 | 0.75 | 0.7, 0.79 | 8.57E-69 |
| LINC01116 | LOXL1 | 21.9 | 368 | 0.75 | 0.7, 0.79 | 9.44E-69 |
| LINC01116 | NKX6-1 | 21.8 | 368 | 0.75 | 0.7, 0.79 | 2.87E-68 |
| LINC01116 | WARS | 21.8 | 368 | 0.75 | 0.7, 0.79 | 4.81E-68 |
| LINC01116 | LRRC17 | 21.7 | 368 | 0.75 | 0.7, 0.79 | 9.42E-68 |
| LINC01116 | ELN | 21.6 | 368 | 0.75 | 0.7, 0.79 | 2.20E-67 |
| LINC01116 | DRD2 | 21.5 | 368 | 0.75 | 0.7, 0.79 | 3.61E-67 |
| LINC01116 | HTR7 | 21.5 | 368 | 0.75 | 0.7, 0.79 | 4.08E-67 |
| LINC01116 | EMX2 | 21.5 | 368 | 0.75 | 0.7, 0.79 | 4.40E-67 |
| LINC01116 | SCN3A | 21.5 | 368 | 0.75 | 0.7, 0.79 | 7.57E-67 |
| LINC01116 | GLI1 | 21.4 | 368 | 0.75 | 0.7, 0.79 | 1.09E-66 |
| LINC01116 | FEZF1 | 21.3 | 368 | 0.74 | 0.69, 0.79 | 2.37E-66 |
| LINC01116 | MYLK2 | 21.3 | 368 | 0.74 | 0.69, 0.79 | 4.25E-66 |
| LINC01116 | PUM3 | 21.2 | 368 | 0.74 | 0.69, 0.78 | 1.00E-65 |
| LINC01116 | MMP13 | 21.2 | 368 | 0.74 | 0.69, 0.78 | 1.25E-65 |
| LINC01116 | TBX5 | 21.1 | 368 | 0.74 | 0.69, 0.78 | 2.84E-65 |
| LINC01116 | SH2B2 | 21 | 368 | 0.74 | 0.69, 0.78 | 9.05E-65 |
| LINC01116 | INSRR | 20.9 | 368 | 0.74 | 0.69, 0.78 | 1.83E-64 |
| LINC01116 | PAMR1 | 20.9 | 368 | 0.74 | 0.69, 0.78 | 2.45E-64 |
| LINC01116 | VWA5B2 | 20.7 | 368 | 0.73 | 0.68, 0.78 | 1.02E-63 |
| LINC01116 | MMP14 | 20.3 | 368 | 0.73 | 0.67, 0.77 | 6.91E-62 |
| LINC01116 | GLT8D2 | 20.3 | 368 | 0.73 | 0.67, 0.77 | 7.95E-62 |
| LINC01116 | HCN4 | 20.1 | 368 | 0.72 | 0.67, 0.77 | 4.70E-61 |
| LINC01116 | CHRM4 | 20.1 | 368 | 0.72 | 0.67, 0.77 | 5.69E-61 |
| LINC01116 | CILP | 20 | 368 | 0.72 | 0.67, 0.77 | 1.13E-60 |
| LINC01116 | ADGRE1 | 20 | 368 | 0.72 | 0.67, 0.77 | 1.17E-60 |
| LINC01116 | COL6A3 | 19.9 | 368 | 0.72 | 0.67, 0.77 | 2.87E-60 |
| LINC01116 | VCL | 19.8 | 368 | 0.72 | 0.67, 0.76 | 4.28E-60 |
| LINC01116 | ADAM23 | 19.7 | 368 | 0.72 | 0.66, 0.76 | 1.14E-59 |
| LINC01116 | GLI3 | 19.7 | 368 | 0.72 | 0.66, 0.76 | 1.32E-59 |
| LINC01116 | CLDN11 | 19.6 | 368 | 0.71 | 0.66, 0.76 | 6.73E-59 |
| LINC01116 | GALNS | 19.5 | 368 | 0.71 | 0.66, 0.76 | 7.95E-59 |
| LINC01116 | JPH3 | 19.4 | 368 | 0.71 | 0.66, 0.76 | 2.54E-58 |
| LINC01116 | TGFB3 | 19.3 | 368 | 0.71 | 0.65, 0.76 | 7.17E-58 |
| LINC01116 | APCDD1L | 19.3 | 368 | 0.71 | 0.65, 0.76 | 7.35E-58 |
| LINC01116 | MMP2 | 19.2 | 368 | 0.71 | 0.65, 0.75 | 2.67E-57 |
| LINC01116 | SERPINH1 | 19.2 | 368 | 0.71 | 0.65, 0.75 | 2.78E-57 |
| LINC01116 | NUDT10 | 19.1 | 368 | 0.71 | 0.65, 0.75 | 3.32E-57 |
| LINC01116 | TNC | 19.1 | 368 | 0.71 | 0.65, 0.75 | 5.89E-57 |
| LINC01116 | CHSY1 | 19.1 | 368 | 0.71 | 0.65, 0.75 | 6.39E-57 |
| LINC01116 | IL17RD | 18.8 | 368 | 0.7 | 0.64, 0.75 | 8.31E-56 |
| LINC01116 | EMILIN1 | 18.6 | 368 | 0.7 | 0.64, 0.75 | 5.63E-55 |
| LINC01116 | BMP5 | 18.6 | 368 | 0.7 | 0.64, 0.75 | 5.94E-55 |
| LINC01116 | FOXF2 | 18.3 | 368 | 0.69 | 0.63, 0.74 | 8.42E-54 |
| LINC01116 | CHN1 | 18.3 | 368 | 0.69 | 0.63, 0.74 | 1.26E-53 |
| LINC01116 | PTCHD1 | 18.2 | 368 | 0.69 | 0.63, 0.74 | 1.96E-53 |
| LINC01116 | GFRA2 | 18.1 | 368 | 0.69 | 0.63, 0.74 | 5.70E-53 |
| LINC01116 | SLCO1C1 | 18.1 | 368 | 0.69 | 0.63, 0.74 | 6.42E-53 |
| LINC01116 | COL6A1 | 18 | 368 | 0.68 | 0.63, 0.73 | 2.09E-52 |
| LINC01116 | SLC8A3 | 17.9 | 368 | 0.68 | 0.62, 0.73 | 3.96E-52 |
| LINC01116 | SFRP1 | 17.9 | 368 | 0.68 | 0.62, 0.73 | 5.54E-52 |
| LINC01116 | AMPH | 17.9 | 368 | 0.68 | 0.62, 0.73 | 6.70E-52 |
| LINC01116 | AC110814.1 | 17.8 | 368 | 0.68 | 0.62, 0.73 | 9.63E-52 |
| LINC01116 | SIAH3 | 17.8 | 368 | 0.68 | 0.62, 0.73 | 1.81E-51 |
| LINC01116 | GJA1 | 17.7 | 368 | 0.68 | 0.62, 0.73 | 2.62E-51 |
| LINC01116 | STX1A | 17.5 | 368 | 0.67 | 0.62, 0.73 | 1.76E-50 |
| LINC01116 | HOXD10 | 17.2 | 368 | 0.67 | 0.61, 0.72 | 3.57E-49 |
| LINC01116 | QRFPR | 17.1 | 368 | 0.67 | 0.6, 0.72 | 1.20E-48 |
| LINC01116 | SYT6 | 17.1 | 368 | 0.66 | 0.6, 0.72 | 1.61E-48 |
| LINC01116 | LANCL3 | 17 | 368 | 0.66 | 0.6, 0.72 | 2.52E-48 |
| LINC01116 | ITGA8 | 17 | 368 | 0.66 | 0.6, 0.72 | 3.79E-48 |
| LINC01116 | COL1A2 | 16.9 | 368 | 0.66 | 0.6, 0.71 | 1.13E-47 |
| LINC01116 | BCL11A | 16.8 | 368 | 0.66 | 0.6, 0.71 | 1.86E-47 |
| LINC01116 | IL13RA2 | 16.8 | 368 | 0.66 | 0.6, 0.71 | 2.23E-47 |
| LINC01116 | IGSF11 | 16.8 | 368 | 0.66 | 0.6, 0.71 | 2.45E-47 |
| LINC01116 | TMEM35A | 16.8 | 368 | 0.66 | 0.6, 0.71 | 2.47E-47 |
| LINC01116 | ITM2C | 16.7 | 368 | 0.66 | 0.6, 0.71 | 3.49E-47 |
| LINC01116 | FRMPD3 | 16.7 | 368 | 0.66 | 0.59, 0.71 | 4.45E-47 |
| LINC01116 | FRG2 | 16.7 | 368 | 0.66 | 0.59, 0.71 | 6.02E-47 |
| LINC01116 | GPX8 | 16.6 | 368 | 0.65 | 0.59, 0.71 | 1.28E-46 |
| LINC01116 | LSP1 | 16.5 | 368 | 0.65 | 0.59, 0.71 | 4.82E-46 |
| LINC01116 | DPYSL5 | 16.4 | 368 | 0.65 | 0.59, 0.71 | 6.94E-46 |
| LINC01116 | HOXD11 | 16.3 | 368 | 0.65 | 0.58, 0.7 | 1.78E-45 |
| LINC01116 | ACTB | 16.3 | 368 | 0.65 | 0.58, 0.7 | 1.81E-45 |
| LINC01116 | CPZ | 16.3 | 368 | 0.65 | 0.58, 0.7 | 2.34E-45 |
| LINC01116 | IL10 | 16.3 | 368 | 0.65 | 0.58, 0.7 | 2.44E-45 |
| LINC01116 | MFAP4 | 16.2 | 368 | 0.65 | 0.58, 0.7 | 3.95E-45 |
| LINC01116 | VLDLR | 16.2 | 368 | 0.64 | 0.58, 0.7 | 8.96E-45 |
| LINC01116 | GPR55 | 16.1 | 368 | 0.64 | 0.58, 0.7 | 1.09E-44 |
| LINC01116 | FSCN1 | 16.1 | 368 | 0.64 | 0.58, 0.7 | 1.20E-44 |
| LINC01116 | SLC1A5 | 16.1 | 368 | 0.64 | 0.58, 0.7 | 1.22E-44 |
| LINC01116 | AP2M1 | 15.8 | 368 | 0.64 | 0.57, 0.69 | 2.91E-43 |
| LINC01116 | PAX3 | 15.8 | 368 | 0.64 | 0.57, 0.69 | 3.24E-43 |
| LINC01116 | MMP11 | 15.7 | 368 | 0.63 | 0.57, 0.69 | 6.29E-43 |
| LINC01116 | FAM26E | 15.6 | 368 | 0.63 | 0.57, 0.69 | 2.00E-42 |
| LINC01116 | SCN5A | 15.6 | 368 | 0.63 | 0.56, 0.69 | 2.27E-42 |
| LINC01116 | DNLZ | 15.5 | 368 | 0.63 | 0.56, 0.69 | 4.79E-42 |
| LINC01116 | XXYLT1 | 15.4 | 368 | 0.63 | 0.56, 0.69 | 7.53E-42 |
| LINC01116 | MFAP2 | 15.4 | 368 | 0.63 | 0.56, 0.68 | 1.03E-41 |
| LINC01116 | KCNG1 | 15.4 | 368 | 0.63 | 0.56, 0.68 | 1.64E-41 |
| LINC01116 | SLC6A6 | 15.3 | 368 | 0.62 | 0.56, 0.68 | 2.59E-41 |
| LINC01116 | POU3F3 | 15.3 | 368 | 0.62 | 0.56, 0.68 | 3.56E-41 |
| LINC01116 | MRGPRF | 15.3 | 368 | 0.62 | 0.56, 0.68 | 4.54E-41 |
| LINC01116 | LDLRAD3 | 15.3 | 368 | 0.62 | 0.56, 0.68 | 4.56E-41 |
| LINC01116 | SRGAP3 | 15.2 | 368 | 0.62 | 0.55, 0.68 | 1.03E-40 |
| LINC01116 | STK32A | 15.1 | 368 | 0.62 | 0.55, 0.68 | 1.29E-40 |
| LINC01116 | PDPN | 15.1 | 368 | 0.62 | 0.55, 0.68 | 1.31E-40 |
| LINC01116 | BNC2 | 15 | 368 | 0.62 | 0.55, 0.68 | 6.12E-40 |
| LINC01116 | AIM2 | 14.9 | 368 | 0.61 | 0.55, 0.67 | 1.05E-39 |
| LINC01116 | ZNF536 | 14.9 | 368 | 0.61 | 0.55, 0.67 | 1.43E-39 |
| LINC01116 | CER1 | 14.9 | 368 | 0.61 | 0.54, 0.67 | 1.69E-39 |
| LINC01116 | RFLNB | 14.8 | 368 | 0.61 | 0.54, 0.67 | 2.07E-39 |
| LINC01116 | S100Z | 14.8 | 368 | 0.61 | 0.54, 0.67 | 2.85E-39 |
| LINC01116 | ERFE | 14.8 | 368 | 0.61 | 0.54, 0.67 | 4.59E-39 |
| LINC01116 | ARHGEF4 | 14.7 | 368 | 0.61 | 0.54, 0.67 | 5.64E-39 |
| LINC01116 | L1CAM | 14.7 | 368 | 0.61 | 0.54, 0.67 | 6.10E-39 |
| LINC01116 | ATP13A2 | 14.7 | 368 | 0.61 | 0.54, 0.67 | 1.21E-38 |
| LINC01116 | ADPGK | 14.7 | 368 | 0.61 | 0.54, 0.67 | 1.31E-38 |
| LINC01116 | RASL12 | 14.6 | 368 | 0.61 | 0.54, 0.67 | 1.99E-38 |
| LINC01116 | FOXD1 | 14.6 | 368 | 0.61 | 0.54, 0.67 | 2.05E-38 |
| LINC01116 | OLFML3 | 14.5 | 368 | 0.6 | 0.53, 0.66 | 4.60E-38 |
| LINC01116 | PURG | 14.5 | 368 | 0.6 | 0.53, 0.66 | 4.63E-38 |
| LINC01116 | PLXND1 | 14.5 | 368 | 0.6 | 0.53, 0.66 | 4.68E-38 |
| LINC01116 | APELA | 14.5 | 368 | 0.6 | 0.53, 0.66 | 7.54E-38 |
| LINC01116 | RORB | 14.4 | 368 | 0.6 | 0.53, 0.66 | 1.04E-37 |
| LINC01116 | TTYH3 | 14.3 | 368 | 0.6 | 0.53, 0.66 | 4.68E-37 |
| LINC01116 | GFPT2 | 14.2 | 368 | 0.6 | 0.53, 0.66 | 6.73E-37 |
| LINC01116 | NES | 14.2 | 368 | 0.6 | 0.53, 0.66 | 6.90E-37 |
| LINC01116 | PRRX1 | 14.2 | 368 | 0.59 | 0.52, 0.66 | 1.03E-36 |
| LINC01116 | CAVIN3 | 14.2 | 368 | 0.59 | 0.52, 0.66 | 1.06E-36 |
| LINC01116 | YKT6 | 14.2 | 368 | 0.59 | 0.52, 0.66 | 1.15E-36 |
| LINC01116 | ST6GALNAC3 | 14.1 | 368 | 0.59 | 0.52, 0.66 | 1.60E-36 |
| LINC01116 | GPR173 | 14.1 | 368 | 0.59 | 0.52, 0.66 | 1.77E-36 |
| LINC01116 | GPR63 | 14.1 | 368 | 0.59 | 0.52, 0.65 | 2.05E-36 |
| LINC01116 | TMEM200C | 14 | 368 | 0.59 | 0.52, 0.65 | 6.85E-36 |
| LINC01116 | TFAP2B | 13.6 | 368 | 0.58 | 0.51, 0.64 | 2.58E-34 |
| LINC01116 | FOXS1 | 13.6 | 368 | 0.58 | 0.5, 0.64 | 3.12E-34 |
| LINC01116 | NEFL | 13.5 | 368 | 0.58 | 0.5, 0.64 | 3.49E-34 |
| LINC01116 | SSPN | 13.5 | 368 | 0.57 | 0.5, 0.64 | 7.59E-34 |
| LINC01116 | FZD2 | 13.4 | 368 | 0.57 | 0.5, 0.64 | 1.20E-33 |
| LINC01116 | NCAM1 | 13.3 | 368 | 0.57 | 0.5, 0.64 | 2.69E-33 |
| LINC01116 | TGFBI | 13.3 | 368 | 0.57 | 0.5, 0.63 | 4.77E-33 |
| LINC01116 | FREM1 | 13.2 | 368 | 0.57 | 0.49, 0.63 | 7.73E-33 |
| LINC01116 | RNF144A | 13.2 | 368 | 0.57 | 0.49, 0.63 | 7.96E-33 |
| LINC01116 | LSAMP | 13.1 | 368 | 0.56 | 0.49, 0.63 | 2.38E-32 |
| LINC01116 | LIMK1 | 13.1 | 368 | 0.56 | 0.49, 0.63 | 2.66E-32 |
| LINC01116 | CD44 | 13.1 | 368 | 0.56 | 0.49, 0.63 | 2.68E-32 |
| LINC01116 | NUAK1 | 12.9 | 368 | 0.56 | 0.48, 0.63 | 9.33E-32 |
| LINC01116 | TPBG | 12.9 | 368 | 0.56 | 0.48, 0.62 | 1.78E-31 |
| LINC01116 | EXT2 | 12.8 | 368 | 0.55 | 0.48, 0.62 | 3.61E-31 |
| LINC01116 | CEP55 | 12.8 | 368 | 0.55 | 0.48, 0.62 | 4.19E-31 |
| LINC01116 | TSHZ3 | 12.7 | 368 | 0.55 | 0.48, 0.62 | 6.30E-31 |
| LINC01116 | SOX18 | 12.7 | 368 | 0.55 | 0.48, 0.62 | 7.11E-31 |
| LINC01116 | LMNB2 | 12.7 | 368 | 0.55 | 0.48, 0.62 | 1.04E-30 |
| LINC01116 | TENM3 | 12.6 | 368 | 0.55 | 0.47, 0.62 | 1.41E-30 |
| LINC01116 | CXCL14 | 12.6 | 368 | 0.55 | 0.47, 0.62 | 1.59E-30 |
| LINC01116 | C10orf55 | 12.5 | 368 | 0.54 | 0.47, 0.61 | 5.43E-30 |
| LINC01116 | LEMD1 | 12.3 | 368 | 0.54 | 0.46, 0.61 | 2.57E-29 |
| LINC01116 | NUP93 | 12.3 | 368 | 0.54 | 0.46, 0.61 | 2.71E-29 |
| LINC01116 | FABP5 | 12.2 | 368 | 0.54 | 0.46, 0.61 | 4.76E-29 |
| LINC01116 | TUBA1C | 12.2 | 368 | 0.54 | 0.46, 0.61 | 6.14E-29 |
| LINC01116 | GNB4 | 12.2 | 368 | 0.54 | 0.46, 0.61 | 6.35E-29 |
| LINC01116 | ZNF521 | 12.2 | 368 | 0.54 | 0.46, 0.6 | 6.91E-29 |
| LINC01116 | PFN2 | 12.1 | 368 | 0.53 | 0.46, 0.6 | 9.07E-29 |
| LINC01116 | PIMREG | 12.1 | 368 | 0.53 | 0.46, 0.6 | 1.18E-28 |
| LINC01116 | PYGO1 | 12.1 | 368 | 0.53 | 0.46, 0.6 | 1.81E-28 |
| LINC01116 | PI15 | 12 | 368 | 0.53 | 0.45, 0.6 | 2.14E-28 |
| LINC01116 | UCHL1 | 12 | 368 | 0.53 | 0.45, 0.6 | 2.16E-28 |
| LINC01116 | NLGN2 | 11.9 | 368 | 0.53 | 0.45, 0.6 | 5.22E-28 |
| LINC01116 | FEV | 11.9 | 368 | 0.53 | 0.45, 0.6 | 1.08E-27 |
| LINC01116 | P3H2 | 11.8 | 368 | 0.52 | 0.45, 0.59 | 1.51E-27 |
| LINC01116 | MEOX1 | 11.8 | 368 | 0.52 | 0.45, 0.59 | 1.58E-27 |
| LINC01116 | COL5A2 | 11.6 | 368 | 0.52 | 0.44, 0.59 | 1.02E-26 |
| LINC01116 | SULF2 | 11.6 | 368 | 0.52 | 0.44, 0.59 | 1.22E-26 |
| LINC01116 | CACNA1G | 11.5 | 368 | 0.51 | 0.44, 0.59 | 2.02E-26 |
| LINC01116 | IQSEC2 | 11.5 | 368 | 0.51 | 0.43, 0.58 | 3.58E-26 |
| LINC01116 | COL13A1 | 11.4 | 368 | 0.51 | 0.43, 0.58 | 4.40E-26 |
| LINC01116 | PRNP | 11.4 | 368 | 0.51 | 0.43, 0.58 | 4.99E-26 |
| LINC01116 | NBL1 | 11.4 | 368 | 0.51 | 0.43, 0.58 | 7.32E-26 |
| LINC01116 | CRTAM | 11.3 | 368 | 0.51 | 0.43, 0.58 | 9.76E-26 |
| LINC01116 | PRKCQ | 11.3 | 368 | 0.51 | 0.43, 0.58 | 9.98E-26 |
| LINC01116 | ANXA1 | 11.3 | 368 | 0.51 | 0.43, 0.58 | 1.20E-25 |
| LINC01116 | SRPX2 | 11.3 | 368 | 0.51 | 0.43, 0.58 | 1.62E-25 |
| LINC01116 | MAFB | 11.3 | 368 | 0.51 | 0.43, 0.58 | 1.82E-25 |
| LINC01116 | SMOX | 11.2 | 368 | 0.51 | 0.43, 0.58 | 1.96E-25 |
| LINC01116 | FEZ1 | 11.2 | 368 | 0.51 | 0.43, 0.58 | 2.09E-25 |
| LINC01116 | AC007040.2 | 11.1 | 368 | 0.5 | 0.42, 0.57 | 5.28E-25 |
| LINC01116 | RIC1 | 11.1 | 368 | 0.5 | 0.42, 0.57 | 5.41E-25 |
| LINC01116 | HOXC13 | 11.1 | 368 | 0.5 | 0.42, 0.57 | 6.43E-25 |
| LINC01116 | ADAM33 | 11.1 | 368 | 0.5 | 0.42, 0.57 | 6.83E-25 |
| LINC01116 | COL6A2 | 11 | 368 | 0.5 | 0.42, 0.57 | 1.43E-24 |
| LINC01116 | PCDHB10 | 11 | 368 | 0.5 | 0.42, 0.57 | 1.84E-24 |
| LINC01116 | PSAP | 11 | 368 | 0.5 | 0.42, 0.57 | 1.97E-24 |
| LINC01116 | TMEM171 | 11 | 368 | 0.5 | 0.42, 0.57 | 2.10E-24 |
| LINC01116 | FOSL1 | 11 | 368 | 0.5 | 0.42, 0.57 | 2.12E-24 |
| LINC01116 | CBWD1 | 10.9 | 368 | 0.5 | 0.41, 0.57 | 2.53E-24 |
| LINC01116 | GLIS3 | 10.9 | 368 | 0.5 | 0.41, 0.57 | 2.54E-24 |
| LINC01116 | RAET1G | 10.9 | 368 | 0.5 | 0.41, 0.57 | 2.85E-24 |

| Table S2. Cut-off, sensitivity and specificy of 11 molecular | | | |
| --- | --- | --- | --- |
| Variables | Cut-off | Sensitivity | Specificity |
| LINC01116 | 6.258 | 86 | 70.27 |
| SOX2 | 3.704 | 90 | 34.05 |
| BEND6 | 7.444 | 88 | 30.54 |
| TMSB15A | 3.152 | 80 | 60.54 |
| PLAU | 249.1 | 86 | 57.03 |
| OLFML2B | 102.5 | 92 | 87.3 |
| NTNG1 | 1.19 | 42 | 75.68 |
| SLC17A7 | 7.956 | 84 | 42.97 |
| NTRK1 | 3.135 | 88 | 46.22 |
| MRC2 | 599.9 | 82 | 64.32 |
| SLC7A3 | 1.016 | 16 | 92.43 |

Figure S1. Scatter plots of LINC01116 and ten protein-coding genes in HCC using the MEARV database. (A-K): Scatter plots of *LINC01116*, *SOX2*, *BEND6*, *TMSB15A*, *PLAU*, *OLFML2B*, *NTNG1*, *SLC17A7*, *NTRK1*, *MRC2*, and *SLC17A3* in HCC.

Figure S2. Results of the molecular mechanism of *TMSB15A* involved in HCC. (A-H): Gene ontology terms of *TMSB15A* may be involved in HCC; (I-L): KEGG pathways of *TMSB15A* may be involved in HCC.

Figure S3. Validation of differential expressions and diagnostic ROC curves using the Oncomine database. (A-H): Validation of the differential expressions of *TMSB15A*, *PLAU*, *OLFML2B*, and *MRC2* using two Oncomine datasets; (I-O): Validation of the diagnostic ROC curves of *TMSB15A*, *PLAU*, *OLFML2B*, and *MRC2* using two Oncomine datasets.

Figure S1.


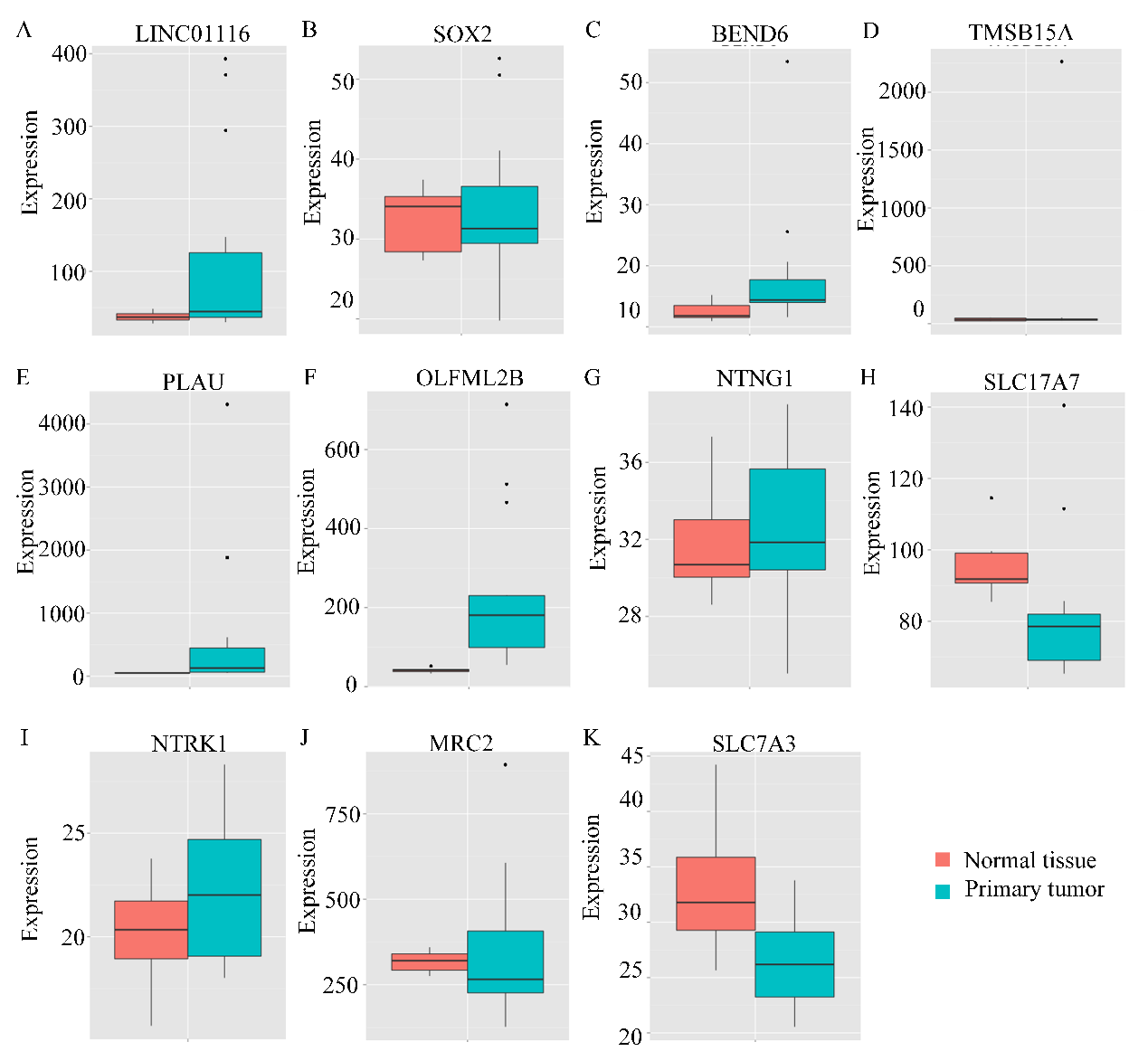


Figure S2.


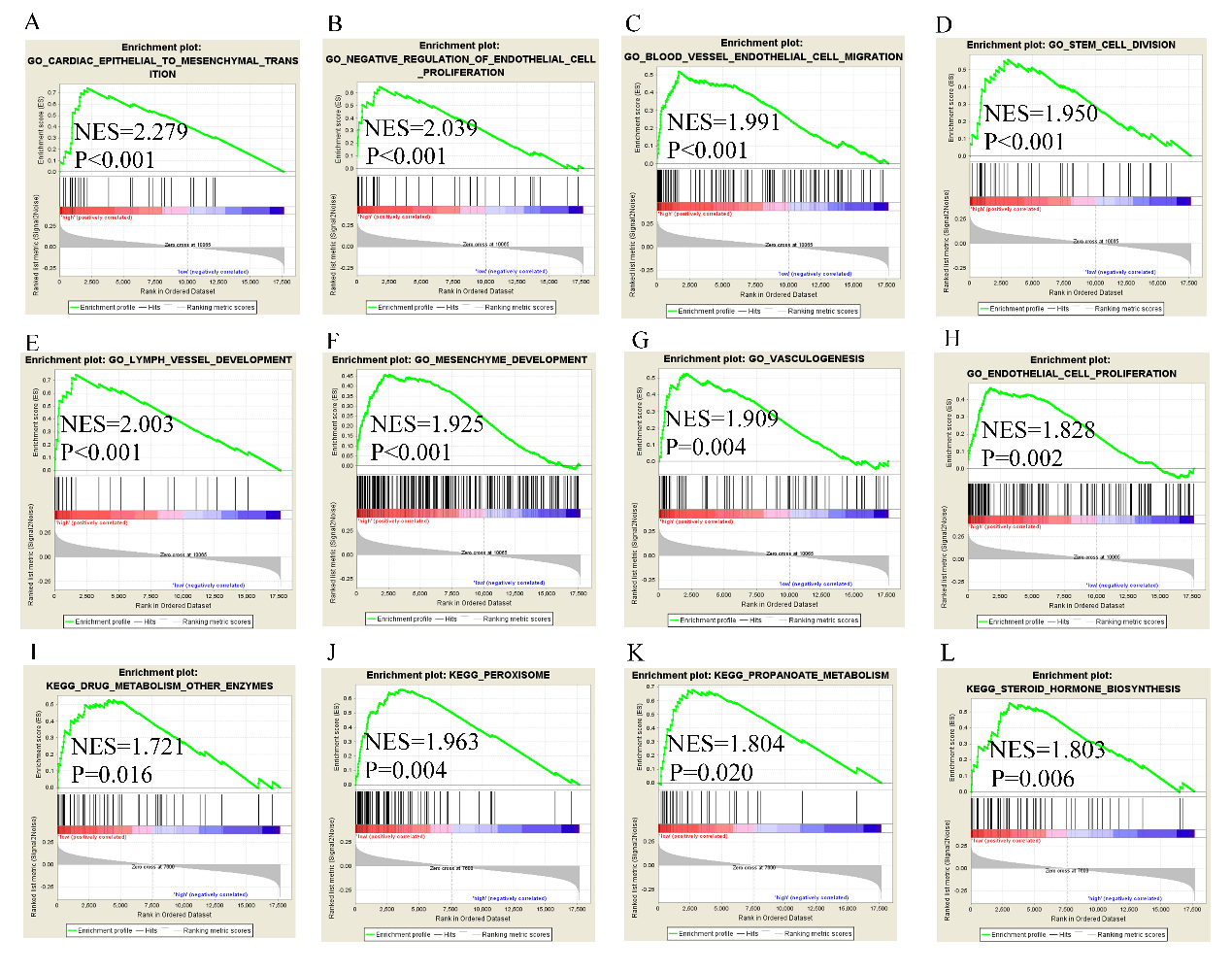


Figure S3.


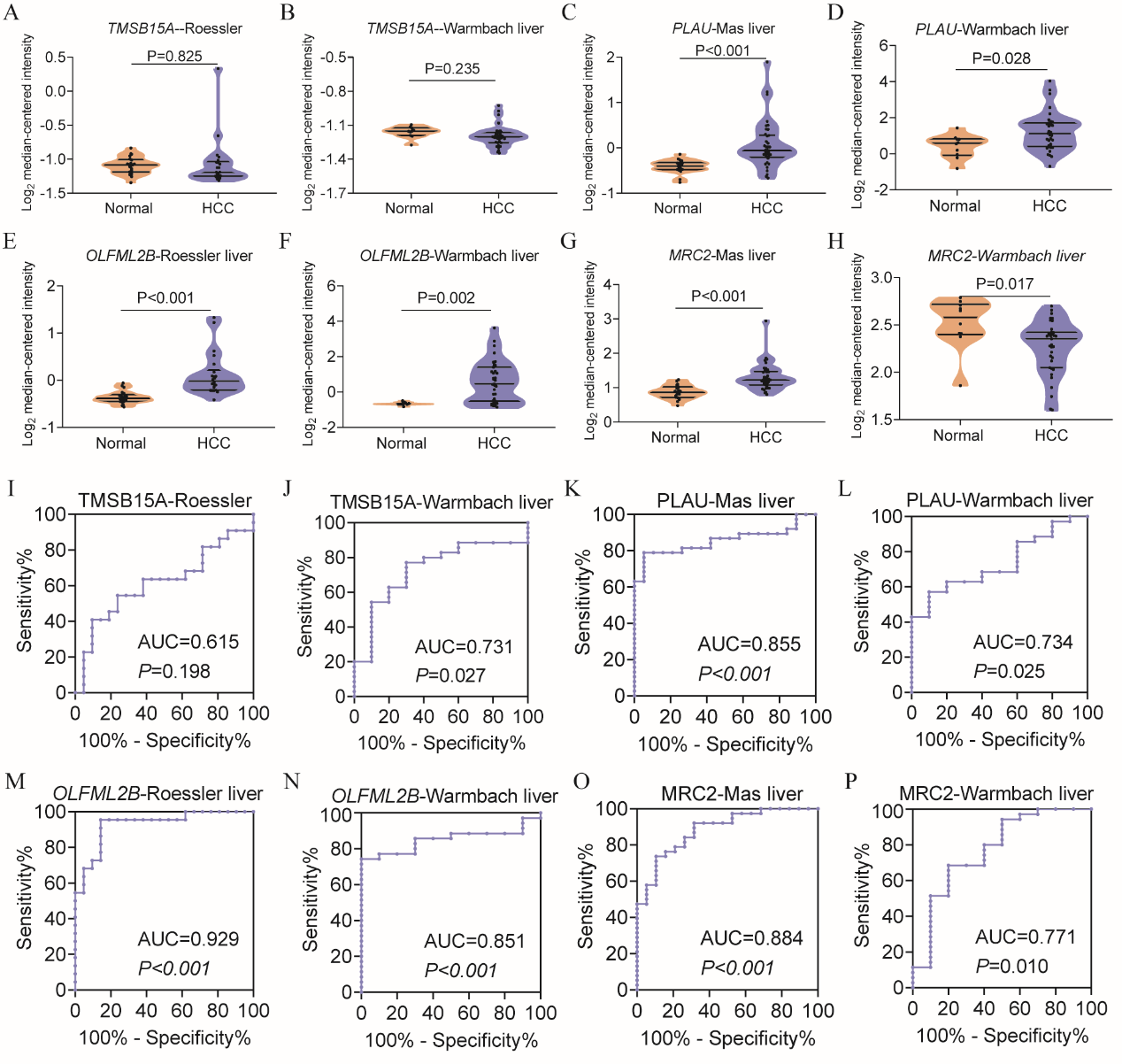

Supplement: Supplementary file 1 — Additional file 1. [file 12876_2023_2827_MOESM1_ESM.docx]
